# Trapping or slowing the diffusion of T cell receptors at close contacts initiates T cell signaling

**DOI:** 10.1073/pnas.2024250118

**Published:** 2021-09-15

**Authors:** Kevin Y. Chen, Edward Jenkins, Markus Körbel, Aleks Ponjavic, Anna H. Lippert, Ana Mafalda Santos, Nicole Ashman, Caitlin O'Brien-Ball, Jemma McBride, David Klenerman, Simon J. Davis

**Affiliations:** ^a^Department of Chemistry, University of Cambridge, Cambridge CB2 1EW, United Kingdom;; ^b^Radcliffe Department of Medicine and United Kingdom Medical Research Council Human Immunology Unit, John Radcliffe Hospital, University of Oxford, Oxford OX3 9DS, United Kingdom;; ^c^School of Physics and Astronomy, University of Leeds, Leeds LS2 9JT, United Kingdom;; ^d^School of Food Science and Nutrition, University of Leeds, Leeds LS2 9JT, United Kingdom

**Keywords:** T cells, receptor triggering, signaling, diffusion analysis, computer simulation

## Abstract

T cell activation is initiated by T cell receptor (TCR) phosphorylation. This requires the local depletion of large receptor-type phosphatases from “close contacts” formed when T cells interact with surfaces presenting agonistic TCR ligands, but exactly how the ligands potentiate signaling is unclear. It has been proposed that TCR ligands could enhance receptor phosphorylation and signaling just by holding TCRs in phosphatase-depleted close contacts, but this has not been directly tested. We devised simple methods to move the TCR in and out of close contacts formed by T cells interacting with supported lipid bilayers (SLBs) and to slow the receptor’s diffusion in the contacts, using a series of anti-CD3ε Fab- and ligand-based adducts of the receptor. TCRs engaging a Fab extended with the large extracellular region of CD45 were excluded from contacts and produced no signaling. Conversely, allowing the extended Fab to become tethered to the SLB trapped the TCR in the close contacts, leading to very strong signaling. Importantly, attaching untethered anti-CD3ε Fab or peptide/MHC ligands, each of which were largely inactive in solution but both of which reduced TCR diffusion in close contacts approximately fivefold, also initiated signaling during cell/SLB contact. Our findings indicate that holding TCRs in close contacts or simply slowing their diffusion in phosphatase-depleted regions of the cell surface suffices to initiate signaling, effects we could reproduce in single-particle stochastic simulations. Our study shows that the TCR is preconfigured for signaling in a way that allows it to be triggered by ligands acting simply as receptor “traps.”

## Significance

Despite the success of T cell–redirecting immunotherapies for blood cancers, toxic, off-target side effects prevent their safe application to other diseases. Part of the challenge lies in our incomplete understanding of T cell receptor (TCR) triggering. Although we know signaling requires TCR phosphorylation, how extracellular TCR/ligand binding produces intracellular phosphorylation is unclear. Using live-cell imaging, we found that just reducing TCR mobility induces T cell signaling. Since native ligands also restrict TCR mobility, our findings suggest that trapping TCRs in tight cell–cell interaction spaces, where large negative-regulatory phosphatases are depleted, might generally initiate T cell signaling. This implies that parameters beyond TCR–ligand affinity could be exploited to fine-tune the specificity and potency of T cell–redirecting agents.

T cells recognize peptide/MHC complexes using receptors comprising obligatory complexes of peptide/MHC-binding αβ TCR and CD3 subunits. The CD3 chains have long cytoplasmic tails that each contain conserved immunoreceptor tyrosine-based activation motifs (ITAMs). TCR engagement with agonist peptide/MHC leads to TCR triggering, which results in the phosphorylation of ITAMs in the CD3 subunits (1, 2). ITAM phosphorylation allows recruitment of the Syk-family kinase ZAP-70, which phosphorylates and recruits a spectrum of downstream targets that drive T cell effector function. Although much is known about the intracellular signaling pathways leading to T cell activation, how the earliest signaling event occurs (i.e., the coupling of ligand binding to receptor phosphorylation) is uncertain (2). Resolving this is key to understanding the remarkable specificity and sensitivity of T cell responses to antigen and important for the design of biologicals used for T cell redirection immunotherapy (3).

The kinetic-segregation (KS) model (4, 5) proposes that when T cells encounter antigen-presenting cells (APCs), “close contacts” are formed by interacting small adhesion proteins such as CD2, creating an intermembrane gap of ∼14 nm (6). This is expected to enhance TCR scanning of the apposing surface for ligands and create a cellular milieu favoring receptor phosphorylation, owing to the local size-dependent exclusion of large phosphatases. In particular, the transmembrane protein tyrosine phosphatase CD45, which has a 21- to 53-nm extracellular domain and acts as a T cell signaling gatekeeper by dephosphorylating TCR-associated CD3 ITAMs (7), becomes passively segregated from close contacts (8–10). The constitutively active tyrosine kinase Lck, which has no extracellular domain and anchors to the inner leaflet of the plasma membrane (11, 12), is then free to phosphorylate TCR/CD3 complexes within close contacts. Based on this physical model, it has been proposed that ligands such as peptide/MHC potentiate signaling by “trapping” TCRs in close contacts. However, this has not been shown.

To test this, we studied the signaling effects of moving TCRs in and out of close contacts. We hypothesized that increasing TCR residence time in close contacts via a reduction in diffusion would suffice to heighten T cell signaling, while excluding TCRs from close contacts would prevent it. Our data supported both these hypotheses. Computational modeling based on experimentally measured parameters further strengthened the case that a “receptor trapping”–based mechanism is sufficient for T cell ligand detection and discrimination.

## Results

### Altered TCR Diffusion Using Fab- and Ligand-Based Adducts.

**Fig. 1.**
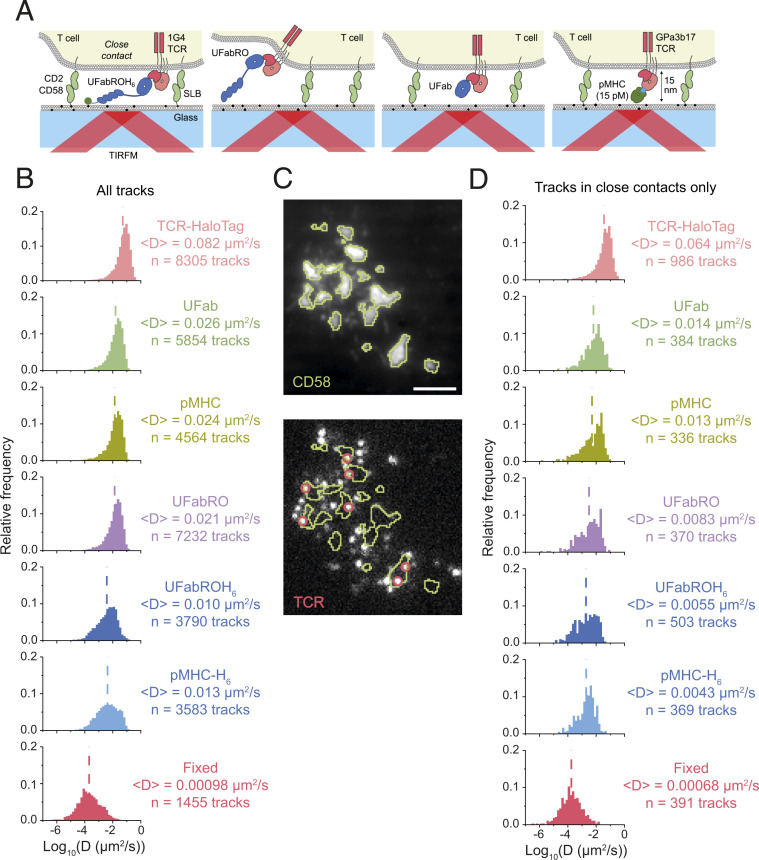
Effects of tethered and untethered Fab- and pMHC-based adducts on TCR diffusion. (*A*) Schematics, drawn to scale in height, showing the UFabROH_6_, UFabRO, UFab, and pMHC adducts bound to TCRs. (*B*) MSD analysis of single-molecule tracks (*n*_TCR–HaloTag_ = 36 cells, *n*_UFab_ = 60 cells, *n*_pMHC_ = 77 cells, *n*_UFabRO_ = 69 cells, *n*_UFabROH6_ = 64 cells, and *n*_pMHC–H6_ = 58 cells). UFab, UFabRO, or UFabROH_6_ were labeled with TMR, pMHC, and pMHC-H_6_ with Alexa-555 and CD58 with Alexa-488. Data are pooled from two independent experiments. Dashed lines indicate average diffusion coefficients. (*C*) Example cell showing outlines (green) of steady-state regions of CD58 accumulation (i.e., close contacts) (*Top*), which were overlaid onto the TCR tracking channel (*Bottom*). Single particles classified as within close contacts are circled (red). (Scale bar, 3 µm.) (*D*) MSD analysis of single-molecule tracks within close contacts (*n*_TCR–HaloTag_ = 31 cells, *n*_UFab_ = 40 cells, *n*_pMHC_ = 55 cells, *n*_UFabRO_ = 60 cells, *n*_UFabROH6_ = 51 cells, and *n*_pMHC–H6_ = 39 cells). MSD analysis of UFabROH_6_-bound TCRs on paraformaldehyde-fixed cells is shown in *B* (all tracks, *n* = 48 cells) and *D* (tracks within close contacts only, *n* = 46 cells) as a measurement of localization precision.

Length differences of ≥5 nm are known to effect the spontaneous segregation of nonbinding molecules from interfaces created by interacting proteins (13–15). Therefore, to reposition the TCR relative to close contacts formed when T cells interacted with supported lipid bilayers (SLBs) used to mimic APC surfaces (16), we attached Fab fragments of the anti-CD3ε antibody UCHT1 that we extended with the extracellular region of CD45RO [length 21 nm (8)], with and without a polyhistidine (6xHis) tag (giving UFabROH_6_ and UFabRO, respectively; Fig. 1*A*). We expected UFabROH_6_ to trap the TCR inside close contacts by binding nickellated lipids in the SLBs and UFabRO to force the receptor out owing to its size. We also prepared native UCHT1 Fab (UFab), expecting that a short (∼7 nm), untethered adduct might reduce TCR mobility without significantly altering its localization (Fig. 1*A*). UFab does not induce any substantial conformational rearrangements within CD3ε (17) and, like other anti-TCR Fab fragments, is believed to be inert in solution (18–20). The Fab adducts were tested on Jurkat T cells expressing the 1G4 TCR (21) and on primary T cells.

To replicate the effects of UFab on TCR diffusion and signaling, we used a second untethered TCR adduct in the form of a soluble, monomeric peptide/MHC (∼6 nm; Fig. 1*A*). To this end, we produced a soluble form of the tumor-associated melanoma antigen gp100 (YLEPGPVTV) complexed with HLA-A*02:01 (hereafter called pMHC), which binds with high affinity (*K*_D_ = 15 pM) to the GPa3b17 TCR (22). We also prepared gp100-pMHC linked to a 6xHis-tag (pMHC-H_6_) which we could attach to nickellated SLBs and use as a mimic of physiological ligand presented on APC membranes. The effects of pMHC and pMHC-H_6_ were tested using Jurkat T cells whose endogenous αβ TCR subunits had been replaced with those of the high-affinity GPa3b17 TCR.

We first confirmed that UFab, UFabRO, UFabROH_6_, pMHC, and pMHC-H_6_ would alter TCR distribution and diffusion. We incubated T cells with small amounts of the tetramethylrhodamine (TMR)- or Alexa-555–conjugated Fab or pMHC adducts and placed them on SLBs that incorporated the synthetic lipid 18:1 DGS-NTA(Ni) to provide attachment sites for polyhistidine-tagged CD58. CD58, the ligand of CD2, was used to facilitate the formation of close contacts. Importantly, NTA(Ni) sites are not fully saturated at equilibrium after incubation with CD58 (23), so unbound sites are available for UFabROH_6_ or pMHC-H_6_ binding during contact formation. Using total internal reflection fluorescence microscopy (TIRFM), we performed single-molecule tracking of the Fab- and pMHC-TCR complexes at the T cell/SLB interface at steady state, 10 min after the cells settled on the SLB (Fig. 1*A* and *SI Appendix*, Supplementary Note S1). We also tracked TCRs labeled on their cytoplasmic domains with HaloTag-JF549 (TCR-HaloTag) in order to measure near-native TCR diffusion.

Using mean square displacement analysis, we found that TCR-HaloTag had an average diffusion coefficient of 0.082 μm^2^/s (Fig. 1*B* and Movie S1), consistent with previous TIRFM tracking measurements reporting 0.08 to 0.09 μm^2^/s for TCRs tagged with fluorescent protein (24). We found that the UFab-bound TCR (UFab/TCR) diffused approximately threefold more slowly, with an average diffusion coefficient of 0.026 μm^2^/s (Fig. 1*B* and Movie S2). pMHC- and UFabRO-bound TCR (pMHC/TCR and UFabRO/TCR) diffusion were slightly slower compared to UFab/TCR, with average diffusion coefficients of 0.024 and 0.021 μm^2^/s, respectively (Fig. 1*B*; UFabRO diffusion is shown in Movie S3). However, the largest reduction in mobility was observed for UFabROH_6_- and pMHC-H_6_–bound TCR (UFabROH_6_/TCR and pMHC-H_6_/TCR), which had average diffusion coefficients of 0.01 and 0.013 μm^2^/s, respectively (Fig. 1*B* and Movie S4). In addition, there was significant overlap between the diffusion coefficient distributions for pMHC-H_6_/TCR or UFabROH_6_/TCR and for TCRs on fixed cells (Fig. 1*B*), indicating that pMHC-H_6_ and UFabROH_6_ effectively immobilized some TCRs. Jump distance (JD) analysis, which aggregates single time step displacements from all trajectories into one distribution for fitting, also supported the trends observed with MSD analysis (*SI Appendix*, Fig. S1*A*). For all conditions, no directed centripetal movement of the TCR or organized clustering was observed (Movies S1–S4), consistent with the cells not being activated by the low density of adducts used for single-molecule tracking.

We also analyzed the subset of trajectories in regions of CD58 accumulation on the SLB, which result from high-affinity CD2/CD58 interactions (25) (Fig. 1 *C* and *D*). These areas of high CD58 density corresponded to regions of CD45 exclusion on T cells (*SI Appendix*, Fig. S2) and are hereafter considered to be synonymous with close contacts. Trajectories analyzed in close contacts, which were defined using a local threshold in the CD58 channel, exhibited the same trends as the global trajectories but with reduced diffusion overall (Fig. 1*D*). The diffusion of TCR-HaloTag was reduced by ∼20%, and UFab/TCR and pMHC/TCR diffused approximately fivefold more slowly than TCR-HaloTag. This is likely due to steric effects, including molecular crowding and a tighter intermembrane space in the close contacts. UFabRO/TCR diffusion in close contacts was more greatly affected than UFab/TCR, pMHC/TCR, and UFabROH_6_/TCR diffusion presumably due to the exclusion of UFabRO/TCR from the close contacts (see *Modulation of TCR Distribution at the T Cell/SLB Interface*), which would have biased the distribution toward slower-diffusing TCRs that did not escape during contact formation (Movie S3).

### Modulation of TCR Distribution at the T Cell/SLB Interface.

**Fig. 2.**
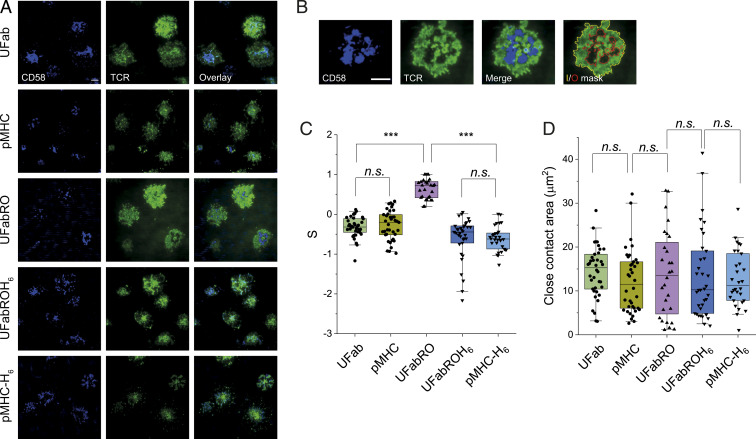
Fab and pMHC adducts trap or exclude TCRs from close contacts. (*A*) Representative fields of view of steady-state contacts and TCR distribution. Cells were incubated with Fab-based adducts or pMHC/pMHC-H_6_, added to CD58-presenting SLBs, and allowed to interact with the SLB for 10 min before imaging of the steady-state contacts and TCR distribution. Dyes used to label proteins were the same as in Fig. 1. Note colocalization of TCR and CD58 for UFab, pMHC, UFabROH_6_, and pMHC-H_6_ incubated cells. In contrast, TCR segregated from regions of CD58 accumulation for UFabRO-treated cells. Also note the central clustering of TCR for UFabROH_6_ and pMHC-H_6_ incubated cells. (Scale bar, 5 μm.) (*B*) Example of a cell incubated with UFabRO displaying close-contact boundaries (red) and cell boundary (yellow) used for TCR segregation level and contact-size quantification. (Scale bar, 5 μm.) (*C*) TCR segregation levels (S) for cells incubated with Fab-based adducts or pMHC/pMHC-H_6_ (*n*_UFab_ = 38 cells, *n*_pMHC_ = 39 cells, *n*_UFabRO_ = 28 cells, *n*_UFabROH6_ = 35 cells, and *n*_pMHC–H6_ = 30 cells). Data are pooled from two independent experiments. (*D*) Close-contact areas of cells analyzed in *C*. Boxplot lines indicate median, 25th and 75th percentile, and fifth and 95th percentile. For TCR segregation and close-contact areas, *P* values were determined using two-sided two sample *t* tests, respectively, equal variance not assumed; *** < 0.001; *n.s.*, not significant.

**Fig. 3.**
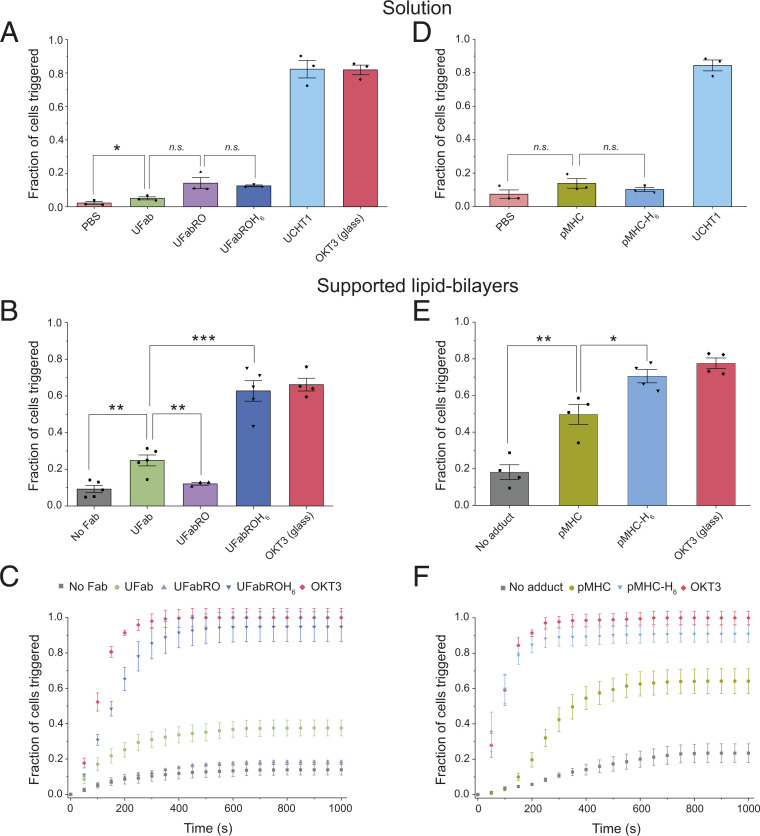
T cells with trapped or slowed TCRs produce calcium signaling. (*A*) Fractions of cells triggered by monomeric Fab-based adducts in solution. Cells were first allowed to settle onto POPC SLBs not presenting CD58, after which UFab, UFabRO, UFabROH_6_, UCHT1 antibody, or PBS only was spiked into the SLB droplet while imaging. The fraction of cells that triggered afterward was measured. Cells were also placed onto OKT3 coated glass as a positive control. Some cells were perturbed and resuspended during the Fab spike, and those that recontacted the SLB after 300 s were excluded since triggering could have occurred in solution before recontact. Error bars are SEM. *n*_PBS_ = 382, 94, and 298 cells, *n*_UFab_ = 281, 223, and 367 cells, *n*_UFabRO_ = 188, 173, and 260 cells, *n*_UFabROH6_ = 221, 246, and 247 cells, *n*_UCHT1 antibody_ = 190, 172, and 351 cells, and *n*_OKT3_ = 88, 204, and 214 cells. (*B*) Fractions of cells triggered for cells incubated with no Fab, UFab, UFabRO, or UFabROH_6_ before addition to a CD58-presenting SLB; error bars are SEM. *n*_noFab_ = 705, 544, 296, 516, and 364 cells, *n*_UFab_ = 236, 319, 760, 158, and 516 cells, *n*_UFabRO_ = 471, 293, and 444 cells, *n*_UFabROH6_ = 367, 199, 586, 272, and 356 cells, and *n*_OKT3_ = 272, 427, 325, and 506 cells. (*C*) Cumulative distribution of triggering times for cells from *B*. Curves are the average of three to five independent experiments; error bars are SEM. Each curve was normalized to the plateau in the OKT3 positive control curve, which measures the fraction of signaling-capable cells. Cells determined to have triggered in solution before SLB contact were not included in the triggering analysis in *B* and *C* (*SI Appendix*, Fig. S4). (*D*) Fractions of cells triggered by monomeric pMHC and pMHC-H_6_ in solution; error bars are SEM. Analysis was performed as in *A*. *n*_PBS_ = 127, 165, and 60 cells, *n*_pMHC_ = 203, 150, and 110 cells, *n*_pMHC–H6_ = 108, 180, and 28 cells, and *n*_UCHT1 antibody_ = 111, 197, and 118 cells. (*E*) Fractions of cells triggered for cells incubated with no adduct, pMHC, or pMHC-H_6_ before addition to a CD58-presenting SLB; error bars are SEM. Analysis was performed as in *B*. *n*_no adduct_ = 64, 384, 117, and 190 cells, *n*_pMHC_ = 329, 196, 202, and 265 cells, *n*_pMHC–H6_ = 282, 150, 201, and 233 cells, *n*_OKT3_ = 62, 139, 142, and 238 cells. (*F*) Cumulative distribution of triggering times for cells from *E*. Curves were generated as in *C*. *P* values were determined using one-sided two sample *t* tests, equal variance not assumed; * < 0.05, ** < 0.01, and *** < 0.001; *n.s.*, not significant.

We next examined the bulk effects of the Fab adducts and pMHC/pMHC-H_6_ on TCR distribution by labeling the cells with saturating amounts of fluorophore-conjugated Fab and pMHC adducts and imaging the interface after 10 min of cell/SLB interaction (Fig. 2*A*). For each cell, the level of TCR segregation (S) was calculated using the TCR intensities inside and outside the close contacts (an example UFabRO-treated cell is shown in Fig. 2*B*; *Materials and Methods*). S > 0 indicated TCR segregation from contacts, while S < 0 indicated accumulation. For the Fab adducts, we also used dynamic, live-cell video (Movies S5–S7) to analyze presteady state segregation levels and contact sizes (*SI Appendix*, Fig. S3) 100 s after cell/SLB contact, before any significant T cell activation had occurred (see Fig. 3).

We found that UFab/TCR and pMHC/TCR were evenly distributed across the majority of cells, indicating that UFab and pMHC do not force the exclusion of TCRs from close contacts (Fig. 2*C*). In fact, we found S < 0 both at steady state and, for UFab/TCR, 100 s after cell contact with the SLB (*SI Appendix*, Fig. S3*A*), suggesting a degree of accumulation likely due to slower diffusion of the TCR complexes inside versus outside of the contacts. In contrast, the distributions of UFabRO/TCR and CD58 accumulation were anti-correlated (Fig. 2*C*), with S > 0.5. Extended UFabRO/TCR complexes presumably leave the contacts to minimize membrane free-energy (14). For UFabROH_6_/TCR and pMHC-H_6_/TCR, no clear segregation from regions of CD58 accumulation was observed (Fig. 2*C*). Instead, S was substantially lower for UFabROH_6_/TCR and pMHC-H_6_/TCR compared to UFab/TCR and pMHC/TCR. Moreover, for UFabROH_6_/TCR, S was below zero 100 s after cell contact with the SLB (*SI Appendix*, Fig. S3*A*). This suggested that the significantly restrained diffusion of UFabROH_6_/TCR and pMHC-H_6_/TCR complexes prevented their exit from contacts despite, in the case of UFabROH_6_, its extra length. Importantly, for the Fab adducts, we also did not observe any significant differences in the distributions of close contact area at steady state (Fig. 2*D*) or 100 s after surface contact (*SI Appendix*, Fig. S3*B*). These data showed that using UFabRO, UFabROH_6_, and pMHC-H_6_, we could move TCRs in and out of contacts without altering cell/SLB adhesion or contact size, which is likely influenced predominantly by CD2/CD58 densities. Using UFab and pMHC, we could also slow the diffusion of the TCR inside close contacts without engaging the apposing surface.

### T Cell Activation by Fabs and pMHC in Close Contacts.

To test whether TCR trapping in close contacts was sufficient to initiate signaling, we used single-cell calcium flux assays (8) to examine the effects of the Fab adducts and pMHC/pMHC-H_6_ on early T cell signaling. Jurkat T cells expressing a genetically encoded calcium indicator (GCaMP) were imaged in real-time as they contacted SLBs presenting CD58, allowing us to measure the time from initial cell/SLB contact to intracellular calcium influx for each cell (*SI Appendix*, Fig. S4). We first confirmed that the Fab adducts and pMHC/pMHC-H_6_ in solution did not invoke substantial amounts of signaling in T cells placed onto SLBs not presenting CD58 (Fig. 3 *A* and *D*), which indicated that the signaling effects of the adducts would require the formation of T cell/SLB contacts. There was some triggering above the control [phosphate buffered saline (PBS) only] for soluble UFab, but the incidence was low (3 to 6%). In contrast to the Fab and pMHC adducts, whole UCHT1 antibody did not require cell/SLB interactions for activation and produced significant T cell signaling in solution, likely due to TCR crosslinking (26). For each of the calcium assays in which T cells were preincubated with the Fab adducts before addition to CD58-presenting SLBs, we also measured the fraction of cells triggered before SLB contact. Consistent with the data in Fig. 3*A*, this background level of triggering was similarly low for all Fab adducts (*SI Appendix*, Fig. S5).

When T cells incubated with UFabROH_6_ or pMHC-H_6_ were added to CD58-presenting SLBs, we observed very significantly elevated and accelerated T cell calcium fluxes that were comparable to those induced by the glass-immobilized OKT3 antibody control (Fig. 3 *B*, *C*, *E*, and *F*). Cells incubated with pMHC-H_6_ triggered slightly faster than after treatment with UFabROH_6_. This indicated that trapping the TCR in close contacts suffices to initiate T cell signaling. Importantly, when cells were incubated with UFab or pMHC, we also observed substantial increases in the fraction of triggered cells compared to untreated cells (Fig. 3 *B*, *C*, *E*, and *F*). This increase was more pronounced for pMHC compared to UFab. In contrast, UFabRO, which slowed TCR diffusion to a similar extent as UFab and pMHC, did not induce activation. We instead observed calcium-signaling levels similar to those for cells incubated without Fab (Fig. 3 *B* and *C*). Primary human T cells obtained from multiple donors were also tested with the Fab adducts, which recapitulated the observations for Jurkat T cells (*SI Appendix*, Fig. S6).

**Fig. 4.**
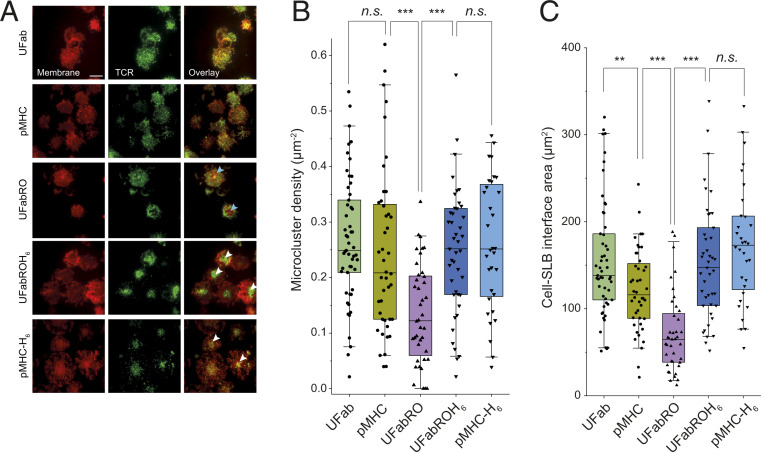
Increased TCR clustering and membrane-spreading correlate with enhanced T cell calcium signaling. (*A*) To complement the calcium signaling–based measure of T cell activation, T cells were incubated with Fab-based adducts or pMHC/pMHC-H_6_ and CellMask Deep Red plasma membrane stain, added to CD58-presenting SLBs, and allowed to interact with the SLB for 10 min before TCR distribution and membrane spreading were imaged using TIRFM. Dyes used to label proteins were the same as in Fig. 1. T cells incubated with UFab or pMHC displayed granular TCR microclusters. T cells incubated with UFabRO did not display such microclustering but exhibited regions of TCR segregation (areas lacking TCR signal but giving CellMask signal, indicated with pale blue arrowheads). T cells incubated with UFabROH_6_ or pMHC-H_6_ displayed large central clusters of TCR (indicated with white arrowheads). (Scale bar, 10 µm.) (*B*) TCR microcluster density for cells incubated with Fab-based adducts or pMHC/pMHC-H_6_. *n*_UFab_ = 53 cells, *n*_pMHC_ = 44 cells, *n*_UFabRO_ = 40 cells, *n*_UFabROH6_ = 43 cells, and *n*_pMHC–H6_ = 32 cells. Data are pooled from two independent experiments. (*C*) Cell/SLB interface area of cells in *B*. Boxplot lines indicate median, 25th and 75th percentile, and 5th and 95th percentile. *P* values were determined using two-sided two sample *t* tests, equal variance not assumed; ** < 0.01, and *** < 0.001; *n.s.*, not significant.

To support these findings, we used TIRFM to image TCR distribution at the cell surface at steady state as a second measure of activation. T cells incubated with UFabROH_6_ or pMHC-H_6_ displayed obvious central regions of TCR accumulation characteristic of immune synapse formation (27) (Fig. 4*A* and *SI Appendix*, Fig. S7 *C* and *E*; also refer to Fig. 2*A*), consistent with the strong calcium-signaling observed for the two adducts (Fig. 3 *B*–*F*). UFab- and pMHC-treated cells did not induce similar centralized TCR clustering, but we did observe a higher degree of TCR microclustering (Fig. 4 *A* and *B*, and *SI Appendix*, Fig. S7 *A* and *D*; also refer to Fig. 2*A*) and cell membrane-spreading (Fig. 4*C*), both indicators of TCR triggering and early T cell activation (28–31), compared to UFabRO-treated cells (Fig. 4*A* and *SI Appendix*, Fig. S7*B*). Correlation analysis confirmed that the apparent accumulation of TCRs into microclusters was not an artifact of membrane height variation in the evanescent field (*SI Appendix*, Fig. S7*F*). UFabROH_6_- and pMHC-H_6_–treated cells also displayed increased TCR microclustering and membrane-spreading (Fig. 4 *B* and *C*). UFabRO-treated cells exhibited no signs of activation, and instead we observed clear contact areas in which there was TCR depletion, presumably due to size-driven exclusion (Fig. 4*A* and *SI Appendix*, Fig. S7*B*). Overall, the imaging data supported the calcium assays. In particular, untethered UFab- and pMHC-induced TCR signaling at close contacts revealed by microcluster formation but not full activation leading to synapse formation, consistent with the calcium flux data showing that UFab and pMHC were moderate stimulators.

Video imaging of Fab adduct-treated T cells as they contacted the SLBs also mirrored the T cell activation data. For example, for cells incubated with UFabROH_6_ we observed rapid cell-spreading with active peripheral lamellipodia and radial TCR transport (Movie S5). Regions of CD58 accumulation also moved centripetally toward the center along with the TCR, as the cells spread. In contrast, cells incubated with UFab did not actively reorganize the membrane (Movie S6), but some TCR microclustering and TCR/CD58 centripetal movement was observed, consistent with the steady-state imaging (Fig. 4). UFabRO-treated cells exhibited none of these features, consistent with the lack of signaling observed in the calcium flux assay (Movie S7). Instead, exclusion of the TCR from areas of CD58 accumulation occurred early during contact formation.

### Quantitative Modeling of TCR Triggering.

**Fig. 5.**
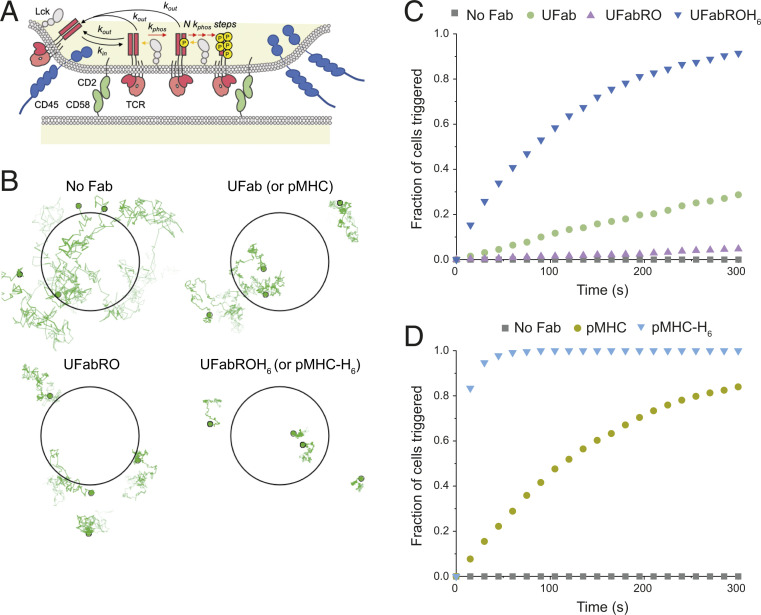
Single-particle stochastic simulations based on experimental data reproduce the T cell signaling behavior observed in experiments. (*A*) Schematic of the KS model of TCR triggering accounting for signaling effects of altered receptor diffusion. TCRs diffusing outside the close contacts formed by the adhesion proteins CD2 and CD58 are maintained in a steady, dephosphorylated state by the action of CD45 phosphatase. TCRs can diffuse into and out of close contacts with rate *k*_in_ and *k*_out_, respectively. Inside the close contact, TCRs can be phosphorylated by Lck (orange arrows) at a rate *k*_phos_. The TCR must acquire a minimum N phosphorylations while inside the close contact to be triggered, and TCRs that diffuse out of the contact before completion of all phosphorylation steps reset to the unphosphorylated state owing to fast CD45-mediated dephosphorylation. (*B*) Snapshots from example simulations showing the effect of the Fab-based adducts or pMHC/pMHC-H_6_ on TCR diffusion and localization within contacts. The close contact (0.22 μm in radius) is indicated by a black circle and TCRs as green circles. Darker shading in tracks corresponds to recent diffusion steps (last 300 steps of each track shown). Only four TCRs are shown, but physiological TCR densities were used for simulations. (*C*) Simulation results (*n* = 1,000 simulations for all curves) showing cumulative fraction of cells triggered versus time using TCR diffusion coefficients and segregation levels taken from experiments with Fab-based adducts (*SI Appendix*, Table S1). (*D*) Same simulations as in *C* with pMHC and pMHC-H_6_.

These experiments showed that trapping the TCR in close contacts (e.g., by using UFabROH_6_ or pMHC-H_6_) suffices to initiate very potent T cell signaling. Remarkably, slowing the diffusion of the TCR in close contacts approximately fivefold (e.g., using UFab or pMHC) also produced clear-cut signaling. We used single-particle stochastic simulations of TCR diffusion in a close contact (Fig. 5 *A* and *B*) to attempt to explain our data quantitatively (*SI Appendix*, Supplementary Note S2 and Table S1). Using Smoldyn (32), we simulated the diffusion of the TCR in and out of close contacts and its phosphorylation by Lck. In simulations carried out using experimentally derived parameters, trends in the levels of TCR triggering matched those in the calcium flux assay (Figs. 3 *C* and *F* and 5 *C* and *D*). In the absence of all ligands, TCR triggering was low due to the fast diffusion of the receptor out of the contact prior to accumulation of enough phosphorylation (Fig. 5 *B*, *Top Left*). Indeed, JD analysis (*SI Appendix*, Fig. S1*A*) confirmed that both the slow and fast populations of TCR-HaloTag had diffusion coefficients above the threshold for signaling identified in the simulations (∼0.0075 μm^2^/s; *SI Appendix*, Fig. S1*B*). In the presence of UFab or pMHC, TCRs resided for longer periods in the contact, with the slow population identified by JD analysis diffusing below the threshold, increasing the likelihood of triggering (Fig. 5 *B*, *Top Right* and *SI Appendix*, Fig. S1*A*). Consistent with the experimental data, pMHC led to more TCR signaling compared to UFab in the simulation due to its slightly slower diffusion compared to UFab (Fig. 1 *B* and *D* and *SI Appendix*, Fig. S1*A*) and the high sensitivity of the model to changes in diffusion in the regime where some TCR particles diffuse below the threshold (*SI Appendix*, Fig. S1*B*). We note that the simulations were more sensitive to changes in diffusion than experiment, which could be due to additional checkpoints between TCR phosphorylation and calcium flux not being modeled.

In contrast to UFab and pMHC, TCR exclusion following UFabRO binding significantly decreased the likelihood of triggering, even though all three adducts had similar mobility (Fig. 5 *B*, *Bottom Left*). Some triggering above native TCR levels was still observed in experiments and simulation likely owing to a reduction in diffusion in opposition to the effect of TCR exclusion. Despite being similar in length to UFabRO, UFabROH_6_ had the effect of anchoring the TCR in the contact, leading to fast and potent receptor triggering (Fig. 5 *B*, *Bottom Right*). pMHC-H_6_ led to even faster signaling presumably due to its smaller dimensions. Therefore, UFab/pMHC and UFabROH_6_/pMHC-H_6_ act as moderate and strong agonists, respectively, by slowing the diffusion or trapping the TCR in close contacts.

**Fig. 6.**
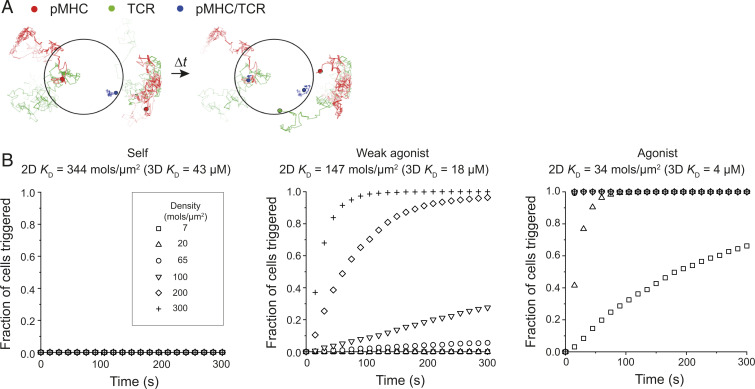
A receptor trapping model of TCR triggering recapitulates T cell signaling specificity and sensitivity. (*A*) Snapshots from example simulations with pMHC. Darker shading in tracks corresponds to more recent diffusion steps (last 300 frames of each track shown). When TCRs (green) and pMHC (red) diffuse within the close contact (black circle), TCR/pMHC complex (blue) formation can occur. Complexes are significantly reduced in mobility (snapshots show a TCR and pMHC binding event within the contact). The snapshot on right is 0.15 s after the snapshot on left in the simulation. (*B*) Simulation results (*n* = 1,000 simulations for all curves) showing cumulative fraction of cells triggered versus time with pMHC of varying 2D affinities and densities (*SI Appendix*, Table S2).

Previous studies indicate that pMHC/TCR binding leads to short periods of receptor immobilization that interrupt free diffusion (33–37). We therefore tested whether a signaling model in which APC-bound pMHC acting as receptor traps within close contacts (Fig. 6*A*) would achieve the specificity and sensitivity of authentic TCR triggering. For this, we performed simulations with pMHC of varying affinities and densities (refer to *SI Appendix*, Supplementary Note S3 for the analytical model and *SI Appendix*, Table S2 for parameters), using two-dimensional (2D) kinetics based on single-molecule fluorescence resonance energy transfer measurements (35). Importantly, we found that T cells relying on a receptor-trapping mechanism exhibited sensitivity and specificity comparable to that observed experimentally (36, 37) (Fig. 6*B*). In terms of sensitivity, >60% of simulated cells responded to a density of 7 agonist ligands/μm^2^ and 100% of the cells triggered within 100 s at 20 pMHC/μm^2^. In comparison, T cell activation measured by calcium flux saturates at agonist densities of 5 to 10 pMHC/μm^2^, indicating that the simulated T cells have comparable sensitivity (36). The simulations also showed that T cell responses were absent for pMHC with affinities ∼10-fold higher than that of the agonist, which is thought to be the typical affinity difference between self and nonself pMHC (37). This indicated that the TCR would also exhibit high levels of signaling specificity in the context of receptor-trapping at close contacts. In response to a weak agonist, T cells exhibited a graded response, with no triggering within the range of conventional agonist pMHC densities (7 to 20 molecules/ μm^2^) but a significant response at self pMHC densities (200 to 300 molecules/μm^2^).

## Discussion

It has been widely reported that CD45 is passively segregated from close contacts formed when T cells interact with artificial and model cell surfaces (8–10, 38–40). Although not yet demonstrated (41), this is also likely to be true in vivo. Not only TCRs but also coreceptors and small adhesion and costimulatory proteins all engage their ligands across an intermembrane distance of ∼14 nm (42), whereas CD45 has an extracellular domain >21 nm in length (8), and size differences of 5 nm are known to secure molecular segregation at model cell interfaces (14, 15). Given the gatekeeping role of CD45 in suppressing untoward phosphorylation (7), receptors residing in close contacts would therefore be more likely to be triggered than those outside, and we have shown elsewhere that if these contacts are large enough, CD45 exclusion alone produces strong TCR triggering (8).

Here, we set out to address the problem of how, in such a context, ligands would heighten TCR signaling, leading to antigen-dependent responses. Our findings that T cell signaling is initiated when TCRs are prevented from leaving close contacts or even if their exit is slowed only fivefold indicate that authentic membrane-tethered ligands would need only to momentarily “trap” the TCR in phosphatase-depleted contacts to initiate strong signaling. Although it has been controversial (43, 44), it is well accepted now that untethered pMHC are largely inactive in free solution, as we have observed. We wish to emphasize, therefore, that the signaling we observe with our untethered UFab and pMHC adducts is wholly contact dependent, since it is contact with the apposing bilayer that likely impedes the diffusion of the adduct-bound TCRs. We could reproduce all our experimental results using simulations that also showed that pMHCs working simply as receptor traps would allow highly sensitive and specific T cell responses. “Diffusion trapping” has been invoked elsewhere to explain TCR triggering but in the context of receptor oligomerization and taking, as its cue, complement fixation by soluble antibody (45).

Previous studies in which forces were supplied to the TCR through experimental pulling or shearing (46, 47) or were generated by actively migrating primary cells (48) have implied that forces applied to the TCR can drive receptor-triggering. These forces are unlikely to be generated in the setting of our experiments as the cells were allowed to passively settle onto SLBs and did not migrate from the original point of contact (Movies S5–S7). Moreover, forces no larger than 2 pN were measured for TCRs interacting with pMHC tethered to SLBs using a fluorescence resonance energy transfer–based force sensor (49). Our observation that signaling is induced by nontethered UFab and pMHC implies that the pulling or shearing forces needed for signaling, if they are required at all, must be extremely weak. In the case of pMHC, we cannot rule out a contribution of allosteric changes priming the TCR for phosphorylation in close contacts, but no significant conformational changes have been observed in structural data (50), and soluble pMHC does not produce calcium fluxes (the present study and refs. 43 and 44). Importantly, our simulations indicate that diffusion effects alone suffice to explain the data.

Our findings are consistent with the work of Aleksic et al. (51), who proposed that fast on-rates allow TCR-pMHC rebinding after initial dissociation, increasing the effective half-life or “confinement time” of binding. A point of departure from this and all other explanations for receptor-signaling is that we are suggesting that bound and unbound forms of the TCR have equivalent signaling activity, as long as they remain in the contact. Our findings are also compatible with work by Lin et al. (52), who showed that T cells respond to rare, long-lived pMHC binding events and not multiple, simultaneous, short-lived TCR interactions. This is consistent with single receptors conducting independent trials of ligand quality. Harder to explain is their finding that multiple, short-binding events occurring sequentially and in close proximity, but unlikely to involve the same TCR, can also initiate signaling. We previously observed surprisingly large effluxes of unengaged receptors from close contacts (53), which can perhaps be attributed to crowding effects (15). One explanation for the findings of Lin et al., therefore, is that rather than sequential binding itself being important for signaling, it is the prevention of TCR escape from close contacts that is responsible for signaling with, perhaps, the last of the bound receptors initiating signaling in their experiments.

The principle that receptor ligands need only to trap receptors in phosphatase-depleted close contacts to initiate signaling, as predicted by the KS model (4, 5), is broadly applicable to ligand-induced signaling by the large set of mostly small receptors phosphorylated by Src family kinases (42). Many of these receptors are monomers and/or monovalent and engage their ligands via rigid-body interactions, foregoing allosteric or other complex structural transformations (54). Our findings also explain the effects of bispecific T cell engagers (BiTEs) and TCR-binding ImmTACs (3) and agonistic antibodies (5) which, in their different ways, are all analogous to the TCR traps we constructed. In further support of receptor-trapping and the need for efficient phosphatase-depletion at close contacts being the key requirements of signaling in such cases, multiple studies have found that chimeric receptors or BiTEs that target epitopes closer to the target cell membrane trigger more effective T cell activation, even if more distal epitopes have higher affinity (55–58). Importantly, this and our previous work (8, 59) have shown that the receptor-trapping mechanism can be incorporated into simulations that predict T cell behavior, which might be helpful for optimizing therapeutics that exploit the receptor-trapping principle.

## Materials and Methods

Details of protein production and labeling, Jurkat T cell transfection, primary T cell isolation, SLB preparation, multicolor TIRFM imaging, and imaging analyses are provided in *SI Appendix*, *Supplementary Materials and Methods*.

### Single-Molecule TCR Tracking.

SLBs presenting CD58 were prepared as described in *SI Appendix*, *Supplementary Materials and Methods*. Jurkat T cells expressing 1G4 TCR or GPa3b17 TCR were labeled substoichiometrically with TMR- or Alexa-555–conjugated UFab (degree of labeling ∼2 dye molecules/protein, as determined by ultra violet-visible light absorption), UFabRO (∼1.5 dye molecules/protein), UFabROH_6_ (∼1.5 dye molecules/protein), pMHC (∼0.7 dye molecules/protein), or pMHC-H_6_ (∼1.0 dye molecules/protein). For labeling, 1 mL of cells at ∼600,000 cells/mL were centrifuged for 90 s, 268 g relative centrifugal force (RCF), resuspended and incubated in 50 μL of ∼1 nM Fab or pMHC/pMHC-H_6_ (in phenol-red free Roswell Park Memorial Institute (RPMI) medium; 1 nM having been determined by titration to be a suitable concentration for single-molecule tracking) for 15 min at 37 °C, and washed twice with 1 mL of PBS buffer. For labeling of T cells expressing TCR-HaloTag fusion protein, the same protocol was used, but cells were incubated with ∼5 nM of JF549 HaloTag ligand. Cells were resuspended in PBS to give a final density of 10,000 cells/μL, pipetted (1 μL) onto the SLBs at room temperature, given 5 min to form close contacts with the SLB, and imaged in TIRFM. For TIRFM imaging of TCRs on fixed cells, cells were labeled with TMR-conjugated UFabROH_6_ using the same protocol. Cells were given 15 min to settle on the SLB, after which they were fixed with 4% paraformaldehyde and 0.02% glutaraldehyde in PBS for 30 min, 4 °C. Cells were then washed three times with PBS and imaged with TIRFM. Image stacks were acquired with an exposure time of 30 ms (33 Hz) or 50 ms (11.76 Hz). The CD58 SLB was imaged before and after tracking with the same exposure time and frame rate.

Single-molecule TCR tracking movies were analyzed in TrackMate (60). Regions of the movie corresponding to cells were first selected manually. For analysis of tracks within close contacts only, averages of the CD58 frames before and after TCR tracking were used to define CD58 accumulation zones, which were selected using Phansalkar local thresholding in Fiji. The CD58 accumulation zones were overlaid onto the TCR channel and used to filter spots. Spots were detected and localized with the Laplacian of Gaussian detector in TrackMate. Spots on the edge of the movies were filtered out from the analysis. Tracks were constructed with the simple Linear Assignment Problem tracker (linking max distance = 3 pixels, gap closing max distance = 3 pixels, and gap-closing max frame gap = 3 frames; 0.107 μm/pixel). Only tracks longer than 20 frames were subsequently used for mean square displacement analysis (*SI Appendix*, *Supplementary Materials and Methods*).

### T Cell Calcium Flux Assay.

To detect T cell signaling, we used a modified version of a standard calcium flux assay (8). A total 1 mL of Jurkat or primary T cells (∼600,000 cells/mL) expressing jGCaMP7 (61) or labeled with Fluo-4, respectively, were centrifuged for 90 s, 268 g RCF, and resuspended in 20 μL of either 1 μM unlabeled Fab adduct or pMHC/pMHC-H_6_ in RPMI, or RPMI alone. Proteins were centrifuged at 17,000 g, 5 min, 4 °C prior to use to remove aggregates. Cells were incubated for 15 min at 37 °C, washed with 1 mL of PBS, and resuspended to ∼10,000 cells/μL in PBS. A total 1 μL of cells was pipetted onto SLBs presenting CD58. Before every calcium flux assay, the health and signaling capacity of the cells were checked by adding untreated cells to OKT3 (full antibody)-coated coverslips (plasma cleaned for 15 min, coated with 0.1 mg/mL OKT3 in PBS for 15 min, and then washed three times with PBS). Image stacks were acquired with exposure time 50 ms (1-Hz frame rate). Refer to *SI Appendix*, *Supplementary Materials and Methods* for calcium flux assay analysis.

### TIRFM Imaging of T cells Treated with the Fab-Based and pMHC/pMHC-H_6_ Adducts.

To image T cell responses in TIRFM, cells were incubated as described for the calcium flux assay, except for being pretreated with 1 μM of labeled UFab, UFabRO, UFabROH_6_, pMHC, or pMHC-H_6_ and 5 μg/mL CellMask Deep Red in phenol-red free RPMI. Cells were pipetted onto SLBs presenting CD58 and allowed to settle for 10 min before imaging using TIRFM. Multiple fields of view were obtained at 50-ms exposure, 11.76 Hz. See *SI Appendix*, *Supplementary Materials and Methods* for microcluster density and cell/SLB interface area analysis.

### Measurement of TCR Segregation and Contact Sizes.

To measure close contact size and TCR segregation levels, Jurkat T cells were stained with the Fab-based adducts or pMHC/pMHC-H_6_ as described for the TIRFM imaging of T cell responses to the adducts. The cells were imaged on SLBs presenting CD58 and after settling on the surface and forming steady-state contacts for 10 min. Image stacks of CD58 and TCR were acquired with an exposure time of 50 ms (11.76 Hz), and 50 to 70 frames averaged in Fiji before analysis. To quantify close-contact size and TCR segregation levels 100 s after initial cell/SLB contact, cells were imaged as they were added to the SLB with an exposure time of 50 ms and frame rate of 0.5 Hz for 20 to 30 min. The TCR and CD58 channels were averaged with 10-frame windows centered on the frame 100 s after initial SLB contact. Initial SLB contact was defined by the first appearance of fluorescence in the TCR channel. The 100-s and steady-state contacts were then analyzed with the bespoke Matlab code described in the *SI Appendix*, *Supplementary Materials and Methods*.

### Stochastic Simulations.

Reaction–diffusion simulations of TCR triggering were run using Smoldyn (version 2.61), a computer program for single-particle spatial stochastic simulations (32). Details of the algorithm used to implement the TCR triggering model can be found in *SI Appendix*, Supplementary Note S2. A bespoke parallel processing Python script (Python 3.8) was used to loop simulations many times for each set of parameters.

## Supplementary Material

Supplementary File

Supplementary File

Supplementary File

Supplementary File

Supplementary File

Supplementary File

Supplementary File

Supplementary File

## Data Availability

All study data are included in the article and/or supporting information.
