# Supplementary material for: Trapping or slowing the diffusion of T cell receptors at close contacts initiates T cell signaling
Source: Proc Natl Acad Sci U S A. 2021 Sep 15;118(39):e2024250118. doi: 10.1073/pnas.2024250118 (PMC8488633; doi:10.1073/pnas.2024250118)
Supplement: Supplementary File [file pnas.2024250118.sapp.pdf]

PNAS
www.pnas.org

Supplementary Information for:

Trapping or Slowing the Diffusion of T-cell receptors at Close Contacts Initiates T-cell Signaling

Kevin Y. Chen[1]*, Edward Jenkins[2]*, Markus Körbel[1], Aleks Ponjavic[1,3,4], Anna H. Lippert[1], Ana Mafalda Santos[2], Nicole Ashman[2], Caitlin O'Brien-Ball[2], Jemma McBride[2], David Klenerman[1], and Simon J. Davis[2]

Correspondence: Simon J. Davis and David Klenerman

Email: simon.davis@imm.ox.ac.uk, dk10012@cam.ac.uk

This PDF file includes:

Supplementary Notes 1 to 3
Supplementary Materials and Methods
Figures S1 to S6
Tables S1 to S2
Legends for Movies S1 to S7
SI References

Other supplementary materials for this manuscript include the following:

Movies S1 to S7

Supplementary Text

Supplementary Note 1: Precision of Single Molecule Tracking Measurements

To measure the localization precision and noise of our single-molecule tracking measurements, we measured the 'diffusion' of TCRs on fixed cells (Fig. 1). As expected, the measurements show that the 'diffusion' of fixed TCRs was significantly slower compared to non-fixed TCRs (Fig. 1B and 1D). JD analysis (Fig. S1), which fits the jump distance distributions with "slow" and "fast" mobility modes, was also used to measure diffusion coefficients. In our JD analysis, a 2-population model was used because a 1-population model was inadequate to describe the jump distance distributions, and a 3-population model did not significantly improve the fit. The contributions of each mobility mode to the fit (f_1 and f_2 in Fig. S1) reflect the proportion of time TCR particles spent in each mobility mode. The JD analysis-based diffusion coefficients reported for fixed TCRs represent the diffusion coefficients obtained from measurement noise. Thus, the localization precision of our measurements based on JD analysis is $\sqrt{4D\Delta t} \cong$ 20-40 nm, where *D* is the diffusion coefficient of fixed TCRs (0.0011-0.0045 μm^2/s, Fig. S1) and Δt is the time between frames (85 ms).

For the non-fixed TCRs, one mobility mode has a diffusion coefficient below 0.005 μm^2/s, which represents either immobility or very slow diffusion, and one mobility mode has a diffusion coefficient greater than 0.01 μm^2/s, which represents faster diffusion that our tracking measurements can resolve. Unlike MSD analysis, JD analysis loses information by aggregating single frame displacements from all single molecule trajectories. JD analysis only obtains information from individual displacements and disregards overall net displacement. Thus, diffusion coefficients below a threshold set by the localization precision (~0.001 μm^2/s) will not be reported in JD analysis. MSD analysis, on the other hand, produces a linear fit of each single molecule trajectory, which can include transitions between slow and fast mobility modes. Although linear fits are not the best model for molecules that switch between sub-diffusive and normal diffusion states, the linear slope can still be seen as an approximate average of the molecule's diffusive behavior throughout the track. Diffusion coefficients smaller than the lower bound for JD analysis are thus possible, as seen in the histograms in Fig. 1.

Supplementary Note 2: Modelling of TCR Triggering

In order to model TCR triggering quantitatively using the collected experimental data on TCR diffusion and segregation, we used the single particle spatial stochastic simulator, Smoldyn (1). Compared to ordinary differential equation-based models, which assume one-pot, well mixed reactions, spatial stochastic simulators can account for 2D membrane confinement, shape and size of the reaction confinement area, and changes in diffusion and mobility of participating receptors and ligands.

During the stochastic simulation, TCR molecules diffuse with Brownian motion within a close contact, i.e. a circle 0.22 μm in radius. A radius of 0.22 μm was chosen based on the sizes of early T-cell microvillar contacts measured in previous studies (2, 3). Of note, the steady-state contact sizes measured in Figure 2 are much larger than initial contacts formed during microvillar search. A growing contact would be more accurate to simulate but is computationally expensive and would not change qualitative trends in the modelling results.

At time *t* = 0, TCR molecules in the unbound, unphosphorylated state are randomly placed in the simulation at density ρ_{TCR} (see Table S1 and Table S2 for the list of simulation parameters). Two populations of TCRs, one faster diffusing and one slower diffusing, were included. Diffusion coefficients and proportions of each population were based on the JD analysis (Fig. S1). For simulation of TCRs bound to UFabRO, the density of TCRs inside the close contact is $1 - p_{seg}$ times the TCR density outside the close contact. p_{seg} was based on measured TCR segregation levels (Fig. 2C). To minimize bias towards expected trends, p_{seg} was conservatively set to 0 for cells incubated with no Fab, UFab, UFabROH$_6$, pMHC, and pMHC-H$_6$, despite some accumulation being observed with those adducts. p_{seg} was conservatively set to 0.5 for UFabRO, despite a higher average segregation being observed in the experiments.

After setting of the initial conditions, the simulation updates the system in discrete time steps of Δt until t_{end} or until a minimum threshold number of TCRs simultaneously attain *N* phosphorylations. Diffusion is simulated by drawing a displacement from a normal distribution and random direction for each

molecule at each time step. Each TCR molecule also has some probability, based on the phosphorylation rate k_{phos}, at each time step of having a phosphate group added if it is within the close contact. However, if the TCR diffuses out of the close contact within the timestep, it immediately resets to the unphosphorylated state. All TCR molecules have a probability 1 of crossing the close contact boundary if they collide with the boundary. However, if TCR is bound to UFab, UFabROH$_6$, pMHC, or pMHC-H$_6$, it has probability < 1 of crossing the boundary from outside to inside ($P_{out\rightarrow in}$). This accounts for the length the adducts add to the TCR extracellular domain. $P_{out\rightarrow in}$ was set to 0.5 for UFabRO/TCR complexes to maintain p_{seg} = 0.5. Since UFabRO and UFabROH$_6$ are similar in size, $P_{out\rightarrow in}$ was set to 0.5 for UFabROH$_6$/TCR complexes as well. Lastly, UFab and pMHC/pMHC-H$_6$ are similar in size and smaller than UFabRO and UFabROH$_6$, so $P_{out\rightarrow in}$ was set to a value intermediate between 0.5 and 1 (0.75) for TCR complexed with UFab or pMHC/pMHC-H$_6$. $P_{in\rightarrow out}$ was set to 1 for all conditions.

For simulations with different affinity pMHC ligands, all rules as described above are the same, except that the TCRs now have some probability of binding pMHC when inside a close contact. Outside of the close contact, the T cell and APC membranes are assumed to be too far apart for TCR and pMHC to bind. For native TCR diffusion, we used the average TCR-HaloTag diffusion coefficient from Fig. 1D. When TCR/pMHC complexes form, TCR diffusion reduces. Previous studies have shown that TCR/pMHC complexes are essentially immobile (4, 5) before becoming centripetally driven by intracellular cytoskeletal forces. Based on single molecule tracking measurements, Axmann *et al.* (4) assumed a TCR/pMHC diffusion coefficient of 0 μm^2/s, which we also adopted.

Supplementary Note 3: Mathematical Analogy between Kinetic Proofreading (KP) and the Kinetic Segregation (KS) Model

In KP, the error rate of triggering can be derived as (6,7)

$$\frac{A_{self}}{A_{agonist}} = e^{-(k_{off,s}-k_{off,a})*\tau} \qquad \text{Eqn. S1}$$

where A_{self} and $A_{agonist}$ are the probabilities that self and agonist pMHC trigger the TCR, respectively, $k_{off,a}$ and $k_{off,s}$ are the off-rates of the agonist and self pMHC, respectively, and τ is the minimum time delay between pMHC/TCR binding and TCR triggering. τ depends on the number of phosphorylation steps required for TCR triggering and the phosphorylation rate. Eqn. S1 displays the hallmark of the classic KP model: for long enough delays (τ = 5-10 s), the error rate is small (<0.001) even for moderate differences (5-10 fold) between the agonist and self pMHC off-rates ($k_{off,s}$ > $k_{off,a}$). In the KS model, the same equation can be written, but with the rate of TCR diffusion out of a close contact zone in lieu of the pMHC/TCR off-rates. Analogous to Eqn. S1, the error rate for the KS model can be derived as

$$\frac{A_{self}}{A_{agonist}} = e^{-(k_{out,s}-k_{out,a})*\tau} \qquad \text{Eqn. S2}$$

where $k_{out,a}$ and $k_{out,s}$ are the rates of TCR diffusion out of a close contact when agonist or self pMHC are present in the contact, respectively, and τ is the time delay between TCR entry into the close contact and TCR triggering (rather than between TCR/pMHC association and TCR triggering, as in KP). Eqn. S1 and S2 both imply that the TCR can only be phosphorylated in certain states, e.g. when the TCR is bound to pMHC (KP model) or when the TCR is in a close contact (KS model). In addition, the equations imply that the TCR resets to the basal state with no phosphorylation after either TCR unbinding (KP model) or diffusion out of a close contact (KS model). In KP, TCR triggering probability only depends on pMHC-TCR affinity. In the KS model, triggering probability depends on the TCR residence time in a close contact, which depends on pMHC/TCR affinity (pMHC trap TCRs inside close contacts), contact size, and TCR and pMHC diffusion coefficients. Because the affinity of self pMHC is less than that of agonist pMHC, TCRs diffusing in a close contact with self pMHC will spend less time in the contact compared to TCRs diffusing in a close contact with agonist pMHC. Thus, $k_{out,s}$ > $k_{out,a}$. Based on these equations, reducing the TCR diffusion coefficient is predicted to increase TCR triggering probability in the KS model, but not in the classic KP model (the triggering probability in the classic KP model only depends on the intrinsic k_{off}).

Supplementary Materials and Methods

CD58, pMHC, ICAM-1, and Fab Production and Labelling

CD58 and ICAM-1 were produced as previously described (8). For creating UFabRO and UFabROH$_6$, UCHT1 variable domain sequences were obtained from the literature (9). Both the V$_H$ and V$_L$ domains were cloned into respective pOPINVH and pOPINVL backbones. pOPINVH was modified to express the extracellular region of CD45RO (aa26-33 linked to aa195-577, UniProtKB P08575) attached to the C-terminus of CH$_1$ of the heavy chain, giving UFabRO. pOPINVH-UFabRO was further modified with either a His-tag (H$_6$) or C-tag (EDQVDPRLIDGK, for purification with HPC-4 antibody (10) coupled to agarose) at the C-terminus of CD45RO for SLB attachment and/or purification, respectively. For production of proteins, UFabRO or UFabROH$_6$ heavy chain expressing vectors were co-expressed transiently along with pOPINVL expressing the light chain of UCHT1 in HEK 293T cells. After 7-10 days medium was taken from the cultures and proteins purified using either Ni-NTA agarose for UFabROH$_6$ (Qiagen) or anti C-tag antibody beads for UFabRO. For pMHC and pMHC-H$_6$ production, cDNA encoding the extracellular domain of HLA-A2 (residues 25-304, UniProtKB P79603) and β-2-microglobulin (β$_2$M, residues 21-119, UniProtKB P61769) were ligated into pET28a vector (+; kanamycin resistant) for expression in Rosetta 2 (DE3)pLysS competent *E.coli* (Merck). The HLA-A2 gene was modified with/without an H$_6$ tag at the C-terminus to allow interaction with 1,2-dioleoyl-sn-glycero-3 (DGS)-NTA(Ni) containing SLBs. HLA-A2 and β$_2$M were purified from inclusion bodies and folded in the presence of gp100 peptide (YLEPGPVTV; GenScript), as previously described (11).

Monomers of all proteins were isolated by size-exclusion chromatography using an AKTA Pure protein purification system. For pMHC experiments, 100 µg of protein was re-run on an AKTA Pure system on the day of experiment to further ensure that the protein was monomeric. UFab and Gap8.3 anti-CD45 Fab were prepared from purified whole antibody using immobilized papain as directed by the manufacturer (ThermoFisher). Fab digestion and purity were confirmed by size exclusion chromatography. OKT3 antibody was provided by the Human Immunology Unit (WIMM, Oxford). All proteins were snap frozen in dry ice and stored at -80 ˚C until use.

All proteins were labeled *via* incubations with a 10-molar excess of NHS-ester dye in PBS and 100 mM sodium bicarbonate (pH 8.3) for one hour at room temperature in the dark. Labelled protein was then purified from free dye using a 6 kDa size exclusion spin column (Micro Bio-Spin™; columns used as directed by the manufacturer). Protein concentration and degree of labelling were determined by UV-Vis absorption.

T-cell Culture and Lentiviral Transfection

Jurkat T-cell lines were grown at 37 °C in 5% CO_2 in sterile RPMI 1640 medium (Sigma Aldrich) supplemented with 10% fetal calf serum (PAA), 2 mM L-glutamine (Sigma Aldrich), 10 mM HEPES buffer (Sigma Aldrich), 1 mM sodium pyruvate (Sigma Aldrich), and 1% penicillin-streptomycin (Sigma Aldrich). Cells were split to ~100,000 cells/mL with warm medium every 3 days. For imaging, cells were split to ~300,000 cells/mL the day before the experiment.

Lentiviral production and transduction were used to generate stable Jurkat (clone E6-1) cell lines expressing a genetically encoded calcium indicator (jGCaMP7s; Addgene #104463). The jGCaMP7 construct was cloned into the pHR vector lentiviral expression vector (see ref. (8)).

To create the GPa3b17 TCR-expressing T-cell line, endogenous TCRα and TCRβ were ablated from Jurkats using CRISPR-Cas9 (12). The GPa3b17 TCRα and TCRβ chains (13) in pHR vectors were then inserted into the TCRα and TCRβ-deficient Jurkats using lentiviruses.

To produce lentivirus, HEK T-cells were seeded onto 6-well plates 24 hours prior to addition of plasmids. After 24 hours, 0.5 µg of pHR plasmid encoding jGCaMP7, TCRα or TCRβ was co-transfected with vectors expressing lentiviral packaging proteins (0.5 µg of p8.91 and 0.5 µg pMDG). Genejuice (Novagen) was used to transfect the plasmid mixture according to the manufacturer's protocol for adherent cells. Forty-eight hours post-transfection, supernatant was collected, 0.22 µm filtered and added to Jurkat T-cells. Cells were sorted by flow cytometry at least 72 hours post transduction.

Human Primary T-cell Isolation

T cells were obtained from blood leukocyte cones purchased from the NHS Blood and Transplantation Service at the John Radcliffe Hospital, Oxford. $CD8^+$ T cells were isolated by Ficoll-Paque density gradient centrifugation followed by use of the $CD8^+$ T-Cell Isolation Kit (Miltenyi Biotec) according to the manufacturer's protocol. Isolated $CD8^+$ T cells were washed and resuspended in RPMI containing key supplements (indicated above), IL-2 (50 U/ml, PeproTech) and CD3/CD28-coated Human T-Activator Dynabeads (ThermoFisher Scientific). The medium was replaced with medium containing fresh IL-2 every 2-3 days. Dynabeads were removed after day 5, and cells left to further expand for 7 more days. On day 12, aliquots of cells were frozen for future use. 24 hours before use, cells were thawed, washed, and resuspended in RPMI containing supplements and IL-2 (50 U/ml). On the day of use, dead cells were removed using the Dead Cell Removal Kit (Miltenyi Biotec) according to the manufacturer's protocol and placed back into RPMI with supplements and IL-2 (50 U/ml). The cell viability was >95%.

SLB Preparation

SLBs were prepared as previously described (14). Glass coverslips (0.13 mm thickness, 22 by 22 mm, VWR International, UK) were first incubated in Piranha solution (3:1 sulfuric acid:hydrogen peroxide) overnight. Coverslips were then rinsed with MilliQ water (MilliQ, 18.2 MΩ resistance), dried with nitrogen, and argon-plasma cleaned (Argon, PDC-002, Harrick Plasma) for 30 min. Press-to-seal silicon isolators (3 mm in diameter, 1 mm deep, Grace Bio-Labs, Bend, Oregon, USA) were pressed onto the clean coverslips. Five μL of PBS and 5 μL of 1 mg/mL lipid vesicle solution (98 mole % 1-palmitoyl-2-oleoyl-sn-glycero-3-phosphocholine (POPC) and 2 mole % 1,2-dioleoyl-sn-glycero-3 (DGS)-NTA(Ni) (Avanti Polar Lipids, Alabaster USA)) were added to each well and incubated for 30 minutes at room temperature. Wells were then washed five times with PBS. Before adding proteins, the protein solutions were spun at 17,000*g* RCF at 4 °C for 15 min to remove large aggregates. Five μL of H_6-tagged CD58 or ICAM-1 (3-10 ng/μL of protein, labelled with Alexa-488, TMR, or Alexa-647) were then added to each well. At 3 ng/μL with a one-hour incubation, protein density on the SLB was on average 3589 ± 198.9 molecules/μm^2. After the incubation, the wells were washed 5 times with PBS. Before every experiment, proteins on the SLB were imaged in TIRF to check for free protein diffusion and fluidity. This was assessed *via* single molecule imaging and/or fluorescence recovery after photobleaching the SLB.

Multicolour TIRF and Epifluorescence Imaging

Experiments were performed on a bespoke TIRF instrument. For excitation, a 488 nm solid-state laser (Spectra Physics Cyan Laser), 561 nm diode laser (Oxxius Laserbox), and a 638 nm diode laser (Cobolt 06-MLD) were first expanded 44x using two Galilean beam expanders to slightly overfill the back aperture of the TIRF objective (100x Plan Apo TIRF, NA 1.49, oil immersion, Nikon). Depending on the channel being used, the laser beams passed through a 632/22, 561/14, or 482/20 (Semrock) bandpass excitation filter to remove unwanted residual wavelengths. The excitation filters were mounted in an optical filter wheel that rotated filters into position (Cairn OptoSpin). Total internal reflection was achieved by focusing the laser at the back focal plane of the TIRF objective and off-axis so that the emergent beam was collimated and incident at an angle greater than the critical angle (~67 degrees) for a glass-water interface. This generated a ~40 μm diameter TIR footprint with power densities in the range 0.1-1 kW/cm^2 at the coverslip. Emitted fluorescence was collected by the same objective and separated from the excitation light by a quad-edge TIRF dichroic mirror (Di03-R405/488/561/635, Semrock). Depending on the channel being used, the fluorescence emission was filtered using a 676/29, 595/31, or 520/44 bandpass emission filter. The emission filters, mounted in a spinning optical filter wheel (Cairn OptoSpin), were synchronized with the excitation filters to allow three-color fluorescence imaging. For single molecule tracking, a quad-notch filter (NF03-405/488/532/635, Semrock) was added to further reduce background from excitation light. Fluorescence emission was focused onto an EMCCD camera (Andor iXON 897, DU897, Oxford Instruments). For single molecule and close contact imaging, the 1.5x internal magnification was used to achieve a final magnification of 150x. For epifluorescence calcium imaging assays, the same setup was used by focusing the laser at the back focal plane of an air, 0.5 NA, 20x objective (Nikon) on axis. A low power density of ~0.0001 kW/cm^2 was used to avoid laser-induced activation of the cells.

Mean-Squared Displacement and Jump Distance Analysis

MSD analysis of tracks obtained from TrackMate was performed using a bespoke Matlab script based on a previously-described approach (15). For each trajectory, the average of the squared displacements was calculated for multiples of the smallest resolved time interval (Δt, $2\Delta t$, $3\Delta t$, etc.). The MSD plot over $n\Delta t$ was fitted with the linear equation:

$$MSD(n\Delta t) = 4D(n\Delta t) + 4\sigma^2 \qquad \text{Eqn. S3}$$

where σ is the localization precision and D is the two-dimensional diffusion coefficient. Only the first five points ($n = 1\ to\ n = 5$) of the MSD curve for each trajectory was fitted since the minimum trajectory length for analysis was 20 frames. After fitting each trajectory, diffusion coefficients were plotted in a histogram. Trajectories with $D < 0$ were ignored.

For jump distance analysis, the individual displacements from all trajectories were aggregated and binned (number of bins determined by $\sqrt{n}$ where n is the number of displacements). The distribution was then fit with the probability density function:

$$\rho(r, \Delta t) = \frac{r}{2D\Delta t} e^{-\frac{r^2}{4D\Delta t}} \qquad \text{Eqn. S4}$$

where r is the jump distance, Δt is the smallest resolved time interval, and D is the diffusion coefficient. Eqn. S4 was obtained from $\int_0^{2p} \frac{1}{4pD\Delta t} e^{-\frac{r^2}{4D\Delta t}}\, r\, dr\, dq = \frac{r}{2D\Delta t} e^{-\frac{r^2}{4D\Delta t}}\, dr$, where the integrand on the right side of the equation is the probability density for a single Brownian particle diffusing a distance r in a 2D plane in time Δt.

For a multi-population fit, a sum of *m* terms was used:

$$\rho(r, \Delta t) = \sum_{j=1}^{m} f_j * \frac{r}{2D_j\Delta t} e^{-\frac{r^2}{4D_j\Delta t}} \qquad \text{Eqn. S5}$$

where f_j is the contribution of the mobility mode j to the fit and D_j is the respective diffusion coefficient. After fitting, $\sum_{j=1}^{m} f_j \neq 1$, so the fractions reported in Fig. S1 are $\frac{f_j}{\sum_{j=1}^{m} f_j}$.

T-cell Calcium Flux Assay Analysis

Calcium triggering assay movies were analysed with a modified version of a bespoke Matlab code (16) that tracks each cell's position and intensity over time. First, a real-space bandpass filter (noise length scale = 3 pixels, cell length scale = 7-10 pixels) was applied to remove noise and extract signal over background. Peaks corresponding to cells in each frame were located and connected into tracks using a nearest neighbor algorithm (17). Only tracks longer than 210 frames were kept for analysis. The intensity for each cell was averaged in a 3x3 pixel box centered on the peak intensity at each frame. Traces were smoothed using a moving average filter with a 21-frame window. A baseline intensity for each cell was determined by averaging the lower 30% of intensity values for smoothened traces. The baseline was used to find peaks in the calcium signal that had minimum prominence equal to three times the baseline. Finally, the time to triggering for each cell was calculated as $t_{trigg} = t_{spike} - t_{contact}$, where t_{spike} was the time of the first calcium spike and $t_{contact}$ was taken as the start of the track (tracking of cells only began when they contacted the SLB).

Using the smoothened calcium intensity traces from above, the proportion of cells that triggered in solution before contact with the SLB (Fig. S5) was determined using a series of logical filters. First, a cell was counted as "pre-triggered" if $I_{max,cell} \geq 0.6 * I_{max,all}$, where $I_{max,cell}$ is the max intensity in the first 30 frames of the cell trace and $I_{max,all}$ is the max intensity of all frames from all cell traces in the experiment. These cells had a high enough initial intensity compared to the other cells in the experiment to be automatically counted as pre-triggered. If $I_{max,cell} \geq 0.3 * I_{max,all}$, then the cell was counted as pre-triggered if t_{trigg} < 30 sec. Most pre-triggered cells fell in this category because the sharp rise and subsequent gradual drop in intensity from a pre-triggered cell landing in the focal plane

was often detected as an early “triggering” peak (within 30 sec of landing) in the calcium triggering analysis. Some pre-triggered cells had a high baseline intensity and thus had no peaks greater than 3 times the baseline, so a cell that had no triggering peaks was also counted as pre-triggered if $I_{max,cell}$ ≥ 0.3 * $I_{max,all}$ (to pick out cells with high initial intensity as before) and $I_{max,cell}$ ≥ (0.25 * $I_{max,all}$) + baseline (to further filter for cells with an early ‘triggering’ peak above baseline).

Microcluster Density and Cell/SLB Interface Area Analysis

To identify putative TCR microclusters indicative of T-cell activation downstream of TCR triggering, a custom, automated TrackMate Python script was used to analyze steady-state TIRF images of labelled cells. First, the movie stacks (50-70 frames) were averaged in order to identify immobile or slow diffusing puncta over a diffuse background in the TCR channel. Individual cells were manually cropped out of each field of view and analysed with the TrackMate script. In TrackMate, peaks were identified in the TCR channel using the LoG detector (blob diameter 4 pixels) with a low threshold (intensity threshold 30 on the LoG filtered image) to obtain all potential microclusters. Peaks were then filtered using quality, contrast, and estimated diameter thresholds. Otsu thresholding, which aims to minimize the variance within classes, was used to determine the minimum acceptable quality for microcluster spots. Furthermore, spots were filtered with a minimum contrast (Michelson contrast definition) threshold of 0.1 and estimated diameter range window (TrackMate-estimated diameter based on spot contrast) of 2-7 pixels (pixel size of 107 nm yielding 200-700 nm window, which was based on a reported average microcluster diameter of 520 nm (18)). The area for normalization was then obtained from the same averaged TCR channel images through manual outlining of the TCR fluorescence edge. Using the same TIRF movies, the T cell/SLB interface area was also measured for each cell through manual outlining of the cell boundary in the CellMask fluorescence channel and multiplication of the number of pixels in the outlined area by the area/pixel ($0.107^2\ \mu m^2$).

Contact Size and Segregation Analysis

For each cell, the cell boundary was first manually outlined using the TCR channel. The CD58 channel was thresholded using either Phansalkar or MidGrey local thresholding in Fiji to identify close contacts with CD58 accumulation (minimum 20 pixels to be considered a close contact). The close contacts were overlaid onto the TCR channel. The average TCR channel intensity was determined for all regions inside (I_{in}) and outside (I_{out}) the close contacts. Some cells had close contacts with holes, and these holes were excluded from the close contacts. The background intensity $I_{background}$ was determined by averaging the TCR channel intensity of all pixels outside the cell boundary in the TCR channel. The segregation level, S, was determined using the formula $1 - \frac{I_{in} - I_{background}}{I_{out} - I_{background}}$, where S = 0 indicates equal mean TCR intensity inside and outside of close contacts (no segregation) and S = 1 indicates zero mean TCR intensity inside close contacts (high segregation). S was default set to 1 for cells with $(I_{in} - I_{background}) < 0$. S < 0 indicates greater mean TCR intensity inside close contacts compared to outside close contacts (TCR accumulation rather than segregation). Close contact total areas were calculated by multiplying the number of pixels in close contacts by the area/pixel ($0.107^2\ \mu m^2$).

Supplementary Figures

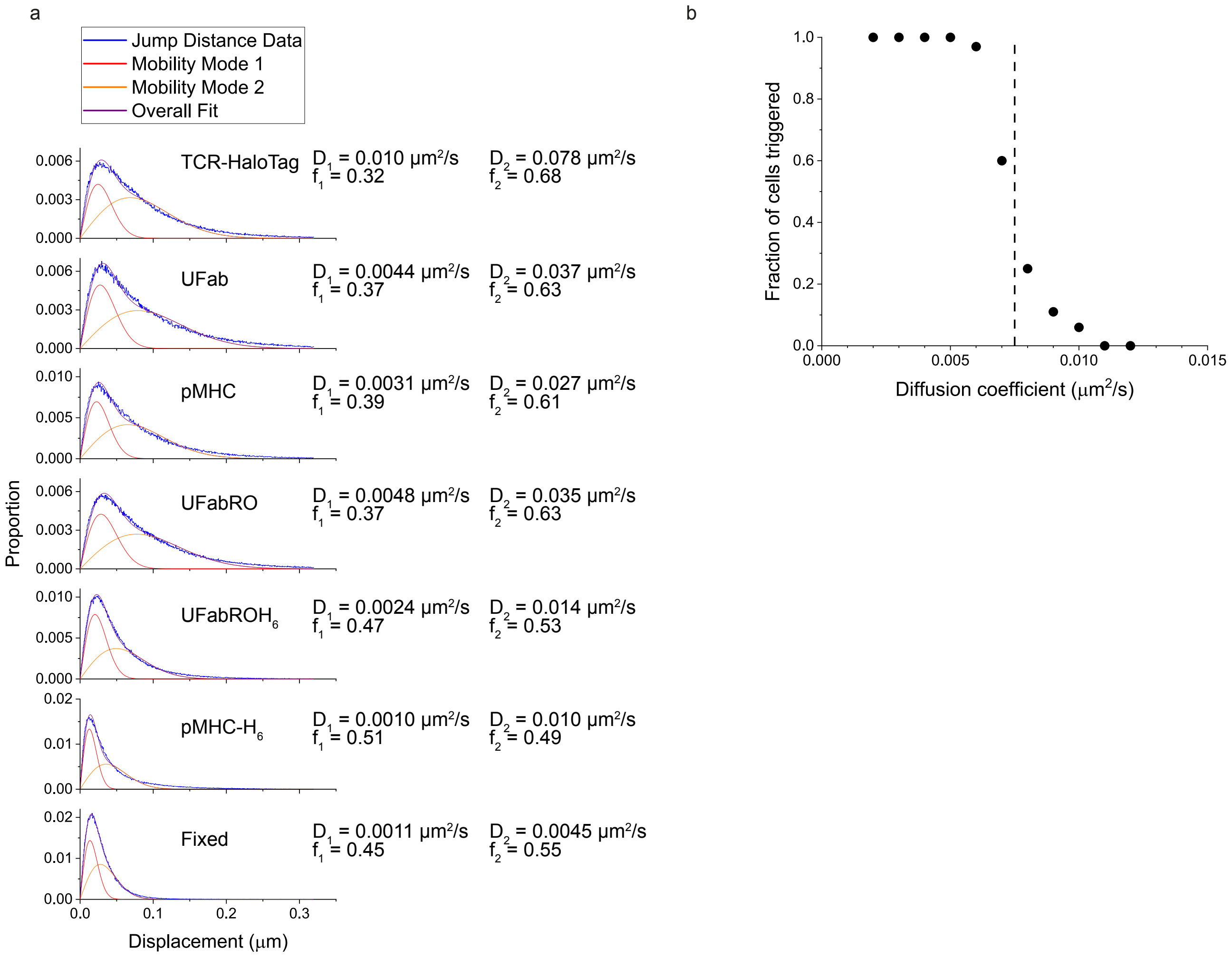

Figure S1. Jump distance analysis of single-molecule TCR tracks. (A) Jump distance analysis of single-molecule TCR tracks from Fig. 1, with two-population fits shown ($n_{TCR-HaloTag} = 332,612$ jumps, $n_{UFab} = 252,182$ jumps, $n_{pMHC} = 184,468$ jumps, $n_{UFabRO} = 321,370$ jumps, $n_{UFabROH6} = 290,173$ jumps, $n_{pMHC-H6} = 230,922$ jumps, $n_{fixed} = 191,875$ jumps). Jump distance stair histogram is shown in blue (y-axis proportion determined by dividing the number of jumps in each bin by total number of jumps), overall fit is shown in purple, and the two individual jump distance distributions that were added together to obtain the overall fit are shown in red and orange. Listed with each curve are the diffusion coefficients characterizing each mobility mode and the weights each mobility mode contributes to the overall fit. See Supplementary Note 1 for discussion of JD vs MSD analysis. (B) Simulation results for the proportion of cells triggering versus TCR diffusion coefficient (*n* = 100 simulations for each diffusion coefficient). All parameters of modelling are the same as those used in Figure 5C and 5D (Table S1), except that the two-population TCR model was reduced to one population (all TCRs diffusing with the same diffusion coefficient, diffusion coefficients tested indicated on the x-axis). Simulation time was also the same as that in Figure 5C and 5D (300 sec). Dashed line indicates the approximate diffusion threshold (0.0075 $\mu m^2/s$) for triggering based on the inflection point of the curve. Note that in panel A, all conditions except TCR-HaloTag have TCRs diffusing at diffusion coefficients below the threshold.

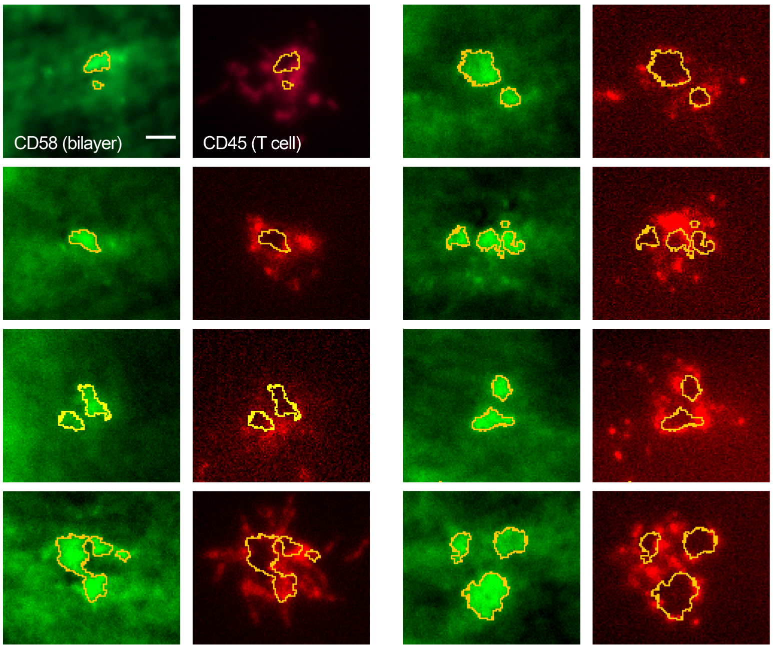

Figure S2. Correspondence of CD58 accumulation in SLBs with CD45 exclusion on T cells. Jurkat T-cells stained with a saturating concentration (1 µM) of Alexa-647-conjugated Gap 8.3 Fab specific for human CD45 were placed onto TMR-conjugated CD58 presenting SLBs and allowed to settle for 10 minutes. Cells and SLB were imaged using TIRFM to check correspondence between CD58 accumulation in the SLB and CD45 exclusion on the cell. CD58 accumulation zones (yellow outlines) were automatically traced as in Figure 2B with local thresholding and the traces were overlaid onto the CD45 channel. Representative sample of 8 cells shown. Almost all CD58 accumulation zones corresponded to sites of CD45 exclusion. Scale bar, 2 µm.

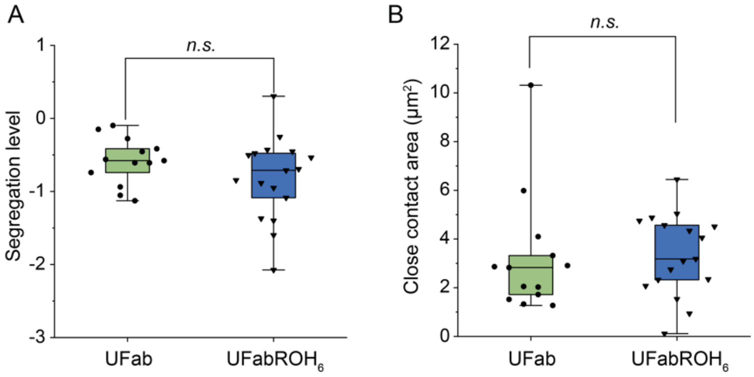

Figure S3. TCR segregation and close contact size of early T cell/SLB contacts. (A) TCR segregation and (B), close contact areas for early contacts 100 sec after T cell/SLB contact (measured in live cell time-lapse movies; see Movies S5-S7). At 100 sec, only 5-30% of cells have triggered (Fig. 3C) and TCR centripetal movement is not underway (Movie S5), thus allowing measurement of TCR segregation and close contact size before significant activation. n_{UFab} = 13 cells, $n_{UFabROH6}$ = 17 cells. Data are pooled from 3 independent experiments. Boxplot lines indicate median, 25th and 75th percentile, and 5th and 95th percentile. Statistical comparisons were made using two-sided two sample *t*-tests, equal variance not assumed.

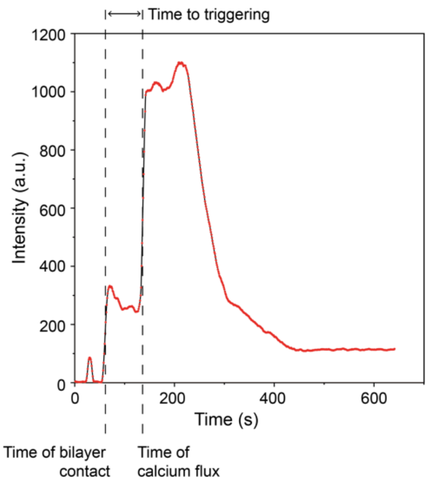

Figure S4. Analysis of calcium signaling. Sample calcium flux trace showing the time of cell contact with the SLB, to time of calcium flux.

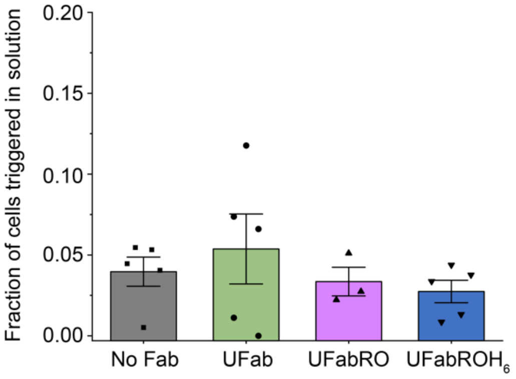

Figure S5. UCHT1 Fabs in solution do not trigger signaling in T cells. Fractions of cells from experiments in Fig. 3B that triggered in solution before SLB contact. Cells were tested for having triggered in solution based on high initial GCaMP fluorescence intensity compared to other cells and the cell's internal baseline, indicating calcium flux before SLB contact (see *SI Materials and Methods*).

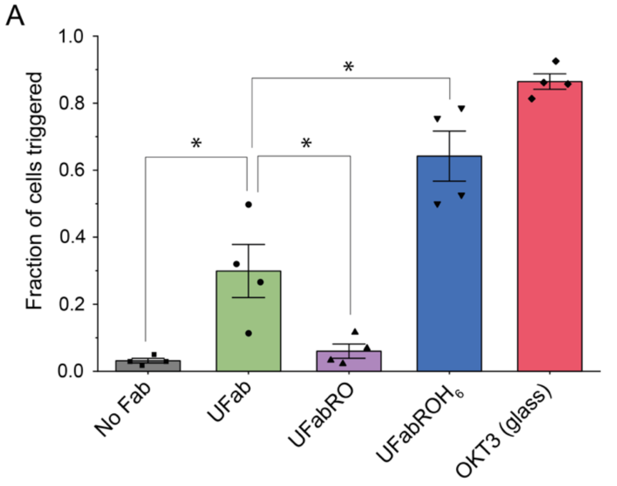

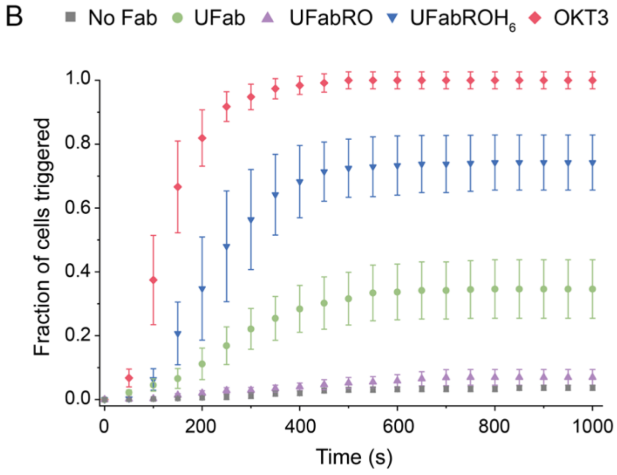

Figure S6. Effects of Fab adducts on signaling by primary human T-cells. (A) Fractions of primary T-cells triggered for cells incubated with no Fab, UFab, UFabRO, or UFabROH6 before addition to a CD58-presenting SLB; error bars are standard error of the mean. $n_{no\ Fab}$ = 458, 229, 68, 102 cells, n_{UFab} = 193, 188, 100, 194 cells, n_{UFabRO} = 86, 268, 160, 336 cells, $n_{UFabROH6}$ = 182, 200, 114, 122 cells, n_{OKT3} = 201, 289, 70, 75 cells. Data was more variable compared to experiments with Jurkat T-cells, possibly due to donor variation among the pooled primary T-cells. (B) Cumulative distribution of triggering times for cells from A. Each curve was normalized to the plateau in the OKT3 positive control curve. Data are analyzed as in Figure 3; error bars are standard error of the mean. P-values were determined using one-sided two sample t-tests, equal variance not assumed; *<0.05.

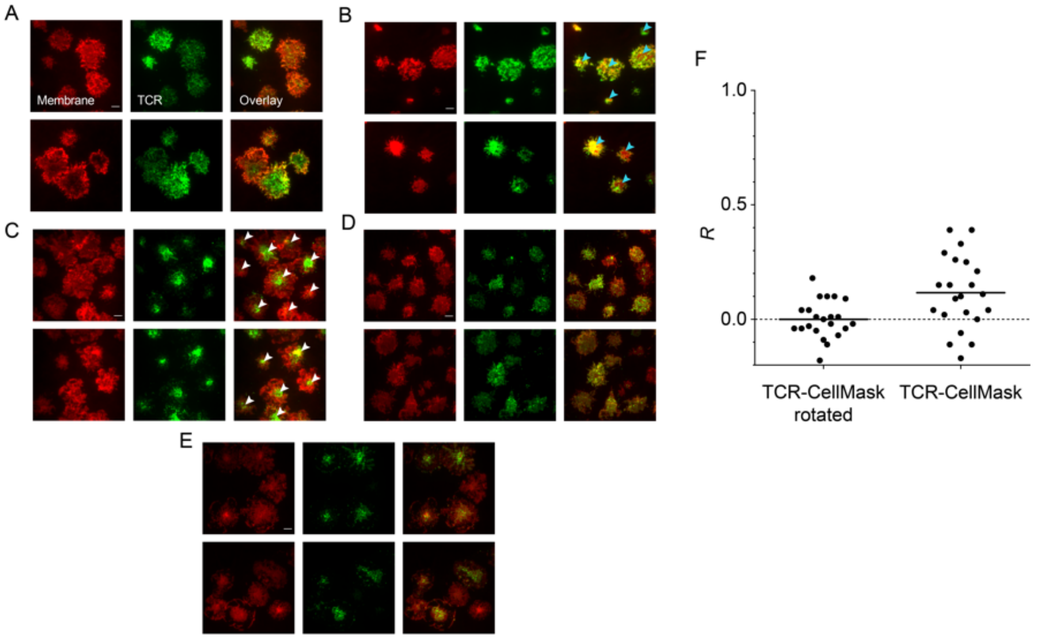

Figure S7. Steady-state TCR distribution on cells incubated with Fab-based adducts. Additional examples of cells from experiments represented in Fig. 4. Cells were incubated with (A) UFab, (B) UFabRO, (C) UFabROH$_6$ (D) pMHC, or (E) pMHC-H$_6$ and CellMask Deep Red plasma membrane stain, added to CD58-presenting SLBs, and allowed to interact with the SLB for 10 minutes before imaging with TIRFM. Each row is a different field of view. Dyes used to label proteins were the same as in Figure 1. Note the granular appearance of TCR in cells incubated with UFab or pMHC. Apparent holes in the TCR channel (some indicated by blue arrowheads) for cells incubated with UFabRO was due to TCR exclusion from close contacts (Fig. 2). Note the central TCR clustering (indicated by white arrowheads for UFabROH$_6$) in cells incubated with UFabROH$_6$ and pMHC-H$_6$. Scale bars, 5 μm. (F), Colocalization between TCR and cell membrane for cells stained with UFab-TMR and CellMask Deep Red (panel A). Pearson's correlation coefficient (19) was used to test whether TCR microclusters correlate with areas of high CellMask intensity. TCR channels were first thresholded using the Otsu method to outline areas corresponding to cell/SLB contact. TCR/cell membrane correlation analysis was then carried out within those areas of interest. R values for the same cells with the TCR channel rotated 90 degrees with respect to the cell membrane channel are provided as a negative control. R values for colocalized species range from 0.6 to 0.8 (20). R for TCR and cell membrane was only ~0.1, indicating that TCR microclusters were not simply the result of uneven CellMask signal. The cells analyzed were a random sample of the UFab-TMR stained cells analyzed in Figure 4B and 4C (*n* = 22 cells). Solid lines indicate means. A dashed line at R = 0 is shown for reference.

Supplementary Tables

Table S1. Parameter values for simulations of TCR triggering with Fab and pMHC adducts.

Parameter	Description	Value	Reference and Comment
r	Close contact radius	0.22 μm	Refs (2, 3)
A_{CCZ}	Close contact area	πr^2	Assume close contact is a circle
r_{sim}	Simulation radius	$2r$ μm	Concentric with close contact
A_{sim}	Simulation area	πr_{sim}^2	Simulation area set as circle
$D_{TCR,slow}$	Unbound TCR diffusion coefficient, slow mobility mode	TCR-HaloTag: 0.010 μm^2/s<br>UFab: 0.0044 μm^2/s<br>UFabRO: 0.0048 μm^2/s<br>UFabROH$_6$: 0.0024 μm^2/s<br>pMHC: 0.0031 μm^2/s<br>pMHC-H$_6$: 0.0010 μm^2/s	Fig. S1, $D_1$of each condition
$D_{TCR,fast}$	Unbound TCR diffusion coefficient, fast mobility mode	TCR-HaloTag: 0.078 μm^2/s<br>UFab: 0.037 μm^2/s<br>UFabRO: 0.035 μm^2/s<br>UFabROH$_6$: 0.014 μm^2/s<br>pMHC: 0.027 μm^2/s<br>pMHC-H$_6$: 0.010 μm^2/s	Fig. S1, $D_2$of each condition
p_{slow}	Proportion of TCRs in slow mobility mode	TCR-HaloTag: 0.68<br>UFab: 0.37<br>UFabRO: 0.37<br>UFabROH$_6$: 0.47<br>pMHC: 0.39<br>pMHC-H$_6$: 0.51	Fig. S1, $f_1$of each condition
p_{fast}	Proportion of TCRs in fast mobility mode	TCR-HaloTag: 0.32<br>UFab: 0.63<br>UFabRO: 0.63<br>UFabROH$_6$: 0.53<br>pMHC: 0.61<br>pMHC-H$_6$: 0.49	Fig. S1, $f_2$of each condition
p_{seg}	Segregation level	TCR-HaloTag: 0<br>UFab: 0<br>UFabRO: 0.5<br>UFabROH$_6$: 0<br>pMHC: 0<br>pMHC-H$_6$: 0	See Supplementary Note 2
$P_{out \to in}$	Probability upon collision of crossing boundary from outside to inside close contact	TCR-HaloTag: 1<br>UFab: 0.75<br>UFabRO: 0.5<br>UFabROH$_6$: 0.5<br>pMHC: 0.75<br>pMHC-H$_6$: 0.75	UFab, UFabRO and UFabROH6 add length to TCR extracellular domain
$P_{in \to out}$	Probability upon collision of crossing boundary from inside to outside close contact	1	No barriers to exit
ρ_{TCR}	Resting T-cell TCR density	100 molecules/μm^2	Ref. (21)
$\rho_{TCR,outside}$	TCR density outside close contact	$\frac{\rho_{TCR} * A_{sim}}{A_{CCZ}(1 - p_{seg}) + (A_{sim} - A_{CCZ}}$	Equation derived to set total number of TCR = $\rho_{TCR} * A_{sim}$
$\rho_{TCR,inside}$	TCR density inside close contact	$\rho_{TCR,outside} * (1 - p_{seg})$	

N	Number of phosphorylation steps	10	10 ITAMS in the TCR (22)
k_{phos}	TCR phosphorylation rate	2.2 s^{-1}	Effective Lck activity in a close contact based on CD45 and Lck densities in the contact (23)
n	Threshold number of TCR with *N* phosphorylations to activate T cell	4	Minimum 4 pMHC-TCR complexes for effective T-cell calcium flux (24)

Table S2. Parameter values for simulations of TCR triggering with different affinity pMHC*.

Parameter	Description	Value	Reference and Comment
D_{TCR}	TCR diffusion coefficient	0.064 μm^2/s	Fig. 1D, average diffusion coefficient for TCR-HaloTag in close contacts
$D_{pMHC-TCR}$	TCR/pMHC complex diffusion coefficient	0	Based on measurements in ref. (4)
p_{seg}	Segregation level	0	Assume no segregation for native TCR
$P_{out \to in}$	Probability upon collision of free TCR crossing boundary from outside to inside close contact	1	No barriers to entry
$P_{in \to out}$	Probability upon collision of free TCR crossing boundary from inside to outside close contact	1	No barriers to exit
ρ_{pMHC}	pMHC density	Varied from 7 to 300 molecules/μm^2	Lower bound: 15-20% of T cells calcium flux by 500 sec at 8.6 molecules/μm^2 (lowest SLB pMHC density in ref. (24))<br><br>Upper bound: 10^5 self pMHC on APCs (25); given average APC diameter of 10-15 µm, self pMHC density is 140-320 molecules/μm^2
$K_{D,3D}$	3D TCR/pMHC dissociation constant	Agonist: 4.26 μM<br>Weak agonist: 18.2 μM<br>Self: 42.6 μM	Based on Table 1 of ref. (26) Taken from median *in situ* (intermolecular FRET measurement) 3D K_D of MCC (IE^k pMHC agonist), T102S (IE^k pMHC weak agonist), and T102G (IE^k pMHC antagonist) peptides
$K_{D.2D}$	2D TCR/pMHC dissociation constant	$\frac{K_{D,3D}}{1.239 * 10^{-7}(M * \mu m^2)}$	Based on calculation in ref. (26)
$k_{off,2D}$	2D TCR-pMHC off rate	Agonist: 0.41 s^{-1}<br>Weak agonist: 1.02 s^{-1}<br>Self: 6.93 s^{-1}	Agonist and weak agonist 2D off rate based on Table 1 of ref. (26); taken from MCC and T102S peptides, respectively<br><br>Self pMHC off rate based on $t_{1/2} \approx 0.1s$ measured in ref. (4) ($k_{off} = \frac{\ln(2)}{t_{1/2}}$)
$k_{on,2D}$	2D TCR/pMHC on rate (2nd order)	$\frac{k_{off,2D}}{K_{D.2D}}$	
$k'_{on,2D}$	2D TCR/pMHC on rate (pseudo 1st-order)	$k_{on,2D} * \rho_{pMHC}$	Pseudo 1st-order on rate used in simulation

*Parameters for simulations with pMHC are identical to those in Table S1 except for those shown in this table.

Supplementary Movies

Movie S1. Diffusion of TCR-HaloTag. Jurkat T-cells expressing TCR-HaloTag fusion bound to JF549-ligand (right channel) were added to Alexa 488-CD58 presenting SLBs (left channel), and the TCRs imaged (200 frames, 33 Hz). The CD58 channel was thresholded to define CD58 accumulation zones (green outline). Magenta circles outline single molecules that were localized within the CD58 accumulation zones during each frame. Tracks shown (30 frame depth) are colored arbitrarily by index. Only tracks >20 frames in length are shown. The cells shown here and in Movies S2-S4 have a higher density of TCR labelling than a typical cell used for single molecule tracking analysis but are displayed to illustrate overall TCR diffusion and localization. Scale bar, 2 μm.

Movie S2. Diffusion of UFab bound TCRs. Jurkat T-cells with TCRs bound to Alexa-555-conjugated UFab (right channel) were added to Alexa 488-CD58 presenting SLBs (left channel), and the TCRs imaged (200 frames, 33 Hz). Close contacts, spots, and tracks are outlined as in Movie S1. Scale bar, 2 μm.

Movie S3. Diffusion of UFabRO bound TCRs. Jurkat T-cells with TCRs bound to TMR-conjugated UFabRO (right channel) were added to Alexa 488-CD58 presenting SLBs (left channel), and the TCRs imaged (200 frames, 12 Hz). Close contacts, spots, and tracks are outlined as in Movie S1. Note that the TCRs are largely excluded from the CD58 accumulation region and diffuse around the edge of the region. Scale bar, 2 μm.

Movie S4. Diffusion of $UFabROH_6$ bound TCRs. Jurkat T-cells with TCRs bound to TMR-conjugated $UFabROH_6$ (right channel) were added to Alexa 488-CD58 presenting SLBs (left channel), and the TCRs imaged (200 frames, 12 Hz). Close contacts, spots, and tracks are outlined as in Movie S1. Compared to Movies S1-S3, TCR diffusion is significantly restricted. Scale bar, 2 μm.

Movie S5. Time lapse of T-cell/SLB interaction with $UFabROH_6$/TCR. Jurkat T-cells with TCR bound to TMR-conjugated $UFabROH_6$ (left channel) were placed onto Alexa 488-CD58 (middle channel) and Alexa 647-ICAM-1 (right channel) presenting SLBs, and the interface was imaged (0.5 Hz). Each channel was bleach corrected (Fiji) and filtered to reduce shot noise (Kalman Filter, Fiji). An outline of the cell lamellipodia can be seen in the TCR and ICAM-1 channel. The cell in the center of the video and two partially cutoff neighboring cells below can be seen pushing against each other as the cells spread. The cells all exhibited similar TCR centripetal movement activity due to activation. Scale bar, 5 μm.

Movie S6. Time lapse of T-cell/SLB interaction with UFab/TCR. Jurkat T-cells with TCRs bound to TMR-conjugated UFab (left channel) were placed onto Alexa 488-CD58 (middle channel) and Alexa 647-ICAM-1 (right channel) presenting SLBs, and the interface was imaged (0.5 Hz). The movie was bleach corrected as in Movie S5. Some TCR microclusters can be seen at 4 minutes after cell contact with the SLB (around 5:50 in the movie), and the microclusters, together with CD58 accumulation and ICAM-1 exclusion zones, exhibited centripetal movement. Scale bar, 5 μm.

Movie S7. Time lapse of T-cell/SLB interaction with UFabRO/TCR. Jurkat T-cells with TCRs bound to TMR-conjugated UFabRO (left channel) were placed onto Alexa 488-CD58 (middle channel) and Alexa 647-ICAM (right channel) presenting SLBs, and the interface was imaged (0.5 Hz). The movie was bleach corrected as in Movie S5. Very little TCR clustering and no centripetal TCR movement occurred. TCR exclusion developed early as regions of CD58 accumulation formed. Scale bar, 5 μm.

Supplementary References

1. S. S. Andrews, Smoldyn: particle-based simulation with rule-based modeling, improved molecular interaction and a library interface. *Bioinformatics* **33**, 710-717 (2016).
2. E. Cai *et al.*, Visualizing dynamic microvillar search and stabilization during ligand detection by T cells. *Science* **356** (2017).
3. P. T. Sage *et al.*, Antigen recognition is facilitated by invadosome-like protrusions formed by memory/effector T cells. *J Immunol* **188**, 3686-3699 (2012).

4. M. Axmann, J. B. Huppa, M. M. Davis, G. J. Schütz, Determination of interaction kinetics between the T cell receptor and peptide-loaded MHC class II via single-molecule diffusion measurements. *Biophysical Journal* **103**, L17-L19 (2012).
5. G. P. O'Donoghue, R. M. Pielak, A. A. Smoligovets, J. J. Lin, J. T. Groves, Direct single molecule measurement of TCR triggering by agonist pMHC in living primary T cells. *eLife* **2**, e00778 (2013).
6. T. W. McKeithan, Kinetic proofreading in T-cell receptor signal transduction. *Proceedings of the National Academy of Sciences* **92**, 5042-5046 (1995).
7. U. Alon, *An introduction to systems biology: design principles of biological circuits* (CRC press, 2019).
8. E. Jenkins *et al.*, Reconstitution of immune cell interactions in free-standing membranes. *J Cell Sci* **132** (2018).
9. K. L. Arnett, S. C. Harrison, D. C. Wiley, Crystal structure of a human CD3-ε/δ dimer in complex with a UCHT1 single-chain antibody fragment. *Proceedings of the National Academy of Sciences* **101**, 16268-16273 (2004).
10. Patent no. WO 95/34652
11. D.N. Garboczi, D.T. Hung, D.C. Wiley. HLA-A2-peptide complexes: refolding and crystallization of molecules expressed in Escherichia coli and complexed with single antigenic peptides. *Proceedings of the National Academy of Sciences* **89**, 3429-33 (1992).
12. Yang H *et al*. HLA-E-restricted, Gag-specific CD8+ T cells can suppress HIV-1 infection, offering vaccine opportunities. *Sci Immunol.* 6:eabg1703 (2021).
13. Liddy, N. Molecular engineering of high affinity T-cell receptors for bispecific therapeutics. Cardiff University (2013).
14. A. Ponjavic *et al.*, Single-molecule light-sheet imaging of suspended T Cells. *Biophys J* **114**, 2200-2211 (2018).
15. L. Weimann *et al.*, A quantitative comparison of single-dye tracking analysis tools using Monte Carlo simulations. *PLOS ONE* **8**, e64287 (2013).
16. V. T. Chang et al., Initiation of T cell signaling by CD45 segregation at 'close contacts'. *Nature Immunology* **17**, 574-582 (2016).
17. J. C. Crocker, D. G. Grier, Methods of digital video microscopy for colloidal studies. *Journal of Colloid and Interface Science* **179**, 298-310 (1996).
18. R. Varma, G. Campi, T. Yokosuka, T. Saito, M. L. Dustin, T cell receptor-proximal signals are sustained in peripheral microclusters and terminated in the central supramolecular activation cluster. *Immunity* **25**, 117-127 (2006).
19. E. M. M. Manders, F. J. Verbeek, J. A. Aten, Measurement of co-localization of objects in dual-colour confocal images. *Journal of Microscopy* **169**, 375-382 (1993).
20. K. W. Dunn, M. M. Kamocka, J. H. McDonald, A practical guide to evaluating colocalization in biological microscopy. *Am J Physiol Cell Physiol* **300**, C723-C742 (2011).
21. B. A. Schodin, T. J. Tsomides, D. M. Kranz, Correlation between the number of T cell receptors required for T cell Activation and TCR–ligand affinity. *Immunity* **5**, 137-146 (1996).
22. J. R. James, Tuning ITAM multiplicity on T cell receptors can control potency and selectivity to ligand density. *Science Signaling* **11**, eaan1088 (2018).
23. R. A. Fernandes *et al.*, A cell topography-based mechanism for ligand discrimination by the T cell receptor. *Proceedings of the National Academy of Sciences* **116**, 14002-14010 (2019).
24. B. N. Manz, B. L. Jackson, R. S. Petit, M. L. Dustin, J. Groves, T-cell triggering thresholds are modulated by the number of antigen within individual T-cell receptor clusters. *Proceedings of the National Academy of Sciences* **108**, 9089-9094 (2011).
25. H. Dumortier *et al.*, Antigen presentation by an immature myeloid dendritic cell line does not cause CTL deletion in vivo, but generates CD8+ central memory-like T cells that can be rescued for full effector function. *J Immunol* **175**, 855-863 (2005).
26. J. B. Huppa *et al.*, TCR-peptide-MHC interactions in situ show accelerated kinetics and increased affinity. *Nature* **463**, 963-967 (2010).
